# Inhibition of pH regulation as a therapeutic strategy in hypoxic human breast cancer cells

**DOI:** 10.18632/oncotarget.17143

**Published:** 2017-04-17

**Authors:** James Meehan, Carol Ward, Arran Turnbull, Jimi Bukowski-Wills, Andrew J. Finch, Edward J. Jarman, Chrysi Xintaropoulou, Carlos Martinez-Perez, Mark Gray, Matthew Pearson, Peter Mullen, Claudiu T. Supuran, Fabrizio Carta, David J. Harrison, Ian H. Kunkler, Simon P. Langdon

**Affiliations:** ^1^ Cancer Research UK Edinburgh Centre and Division of Pathology Laboratory, Institute of Genetics and Molecular Medicine, University of Edinburgh, Edinburgh EH4 2XU, United Kingdom; ^2^ Cancer Research UK Edinburgh Centre, Institute of Genetics and Molecular Medicine, University of Edinburgh, Edinburgh EH4 2XU, United Kingdom; ^3^ IGMM Advanced Imaging Resource, Institute of Genetics and Molecular Medicine, University of Edinburgh, Edinburgh EH4 2XU, United Kingdom; ^4^ School of Medicine, University of St Andrews, Fife KY16 9TF, United Kingdom; ^5^ Department of Neurofarba, Sez. Chimica Farmaceutica e Nutraceutica, University of Florence, Sesto Fiorentino 50019, Italy

**Keywords:** carbonic anhydrase IX, NHE1, V-ATPase, breast cancer, hypoxia

## Abstract

Hypoxic cancer cells exhibit resistance to many therapies. This study compared the therapeutic effect of targeting the pH regulatory proteins (CAIX, NHE1 and V-ATPase) that permit cancer cells to adapt to hypoxic conditions, using both 2D and 3D culture models. Drugs targeting CAIX, NHE1 and V-ATPase exhibited anti-proliferative effects in MCF-7, MDA-MB-231 and HBL-100 breast cancer cell lines in 2D. Protein and gene expression analysis in 2D showed that CAIX was the most hypoxia-inducible protein of the 3 targets. However, the expression of CAIX differed between the 3 cell lines. This difference in CAIX expression in hypoxia was consistent with a varying activity of FIH-1 between the cell lines. 3D expression analysis demonstrated that both CAIX and NHE1 were up-regulated in the hypoxic areas of multicellular tumor spheroids. However, the induction of CAIX expression in hypoxia was again cell line dependent. 3D invasion assays conducted with spheroids showed that CAIX inhibition significantly reduced the invasion of cells. Finally, the capability of both NHE1 and CAIX inhibitors to combine effectively with irradiation was exhibited in clonogenic assays. Proteomic-mass-spectrometric analysis indicated that CAIX inhibition might be combining with irradiation through stimulating apoptotic cell death. Of the three proteins, CAIX represents the target with the most promise for the treatment of breast cancer.

## INTRODUCTION

The unrestricted development of tumors provides momentum for cancer cells to grow and survive in areas further away from blood vessels in regions beyond the effective diffusion distance of oxygen, leading to oxygen deficiency (or hypoxia) in these cells [1]. The tumor vasculature is highly irregular and is often unsuccessful in rectifying this deficit [2], with an estimated 50% of advanced breast cancers containing hypoxic tissue areas [3]. Low oxygen levels reduce the ability of cells to obtain energy through oxidative phosphorylation and cause an increased dependency on glycolysis for the production of energy. Increased glucose consumption through glycolysis leads to the production of H^+^ ions which, if not controlled, can lead to changes in the internal pH of cancer cells. Such changes in intracellular pH (pH_i_) can potentially affect almost all cellular processes [4]. Therefore hypoxic cancer cells, which produce large amounts of H^+^ ions through glycolysis, need to be able to control their pH_i_ to a greater extent than normal cells, or even aerobic cancer cells, to ensure survival within their hostile tumor microenvironment. As such, an adaptive feature of hypoxic cancer cells is the overexpression and/or elevated activity of a number of pH regulating proteins. These proteins include carbonic anhydrase IX (CAIX), Na^+^-H^+^ exchanger 1 (NHE1) and vacuolar H^+^-ATPase (V-ATPase) [5], as illustrated in Supplementary Figure 1.

Each of these proteins contributes to cellular pH homeostasis in different ways. Membrane-permeant CO_2_ is a form in which much acid is removed by tumor cells [6]. This involves the key enzyme CAIX, which facilitates CO_2_ diffusion from cancer cells by catalyzing the extracellular conversion of CO_2_ into HCO_3_^−^ and H^+^, thereby maintaining a steeper efflux gradient for CO_2_ [6]. At the same time, CAIX causes a decrease in extracellular pH (pH_e_) due to the production of H^+^ ions extracellularly. Both NHE1 and V-ATPase differ in their method of pH regulation. NHE1 is extremely sensitive to pH_i_; when pH_i_ drops below a certain level, NHE1 is activated by an internal allosteric H^+^-binding regulatory site, leading to NHE1 extruding one proton in exchange for one Na^+^ ion, thereby alkalinizing pH_i_ and acidifying pH_e_ [7]. Finally, V-ATPases are ATP-dependent H^+^ transporters that transfer protons using the energy released by ATP hydrolysis. They transport H^+^ ions from the cytoplasm to intracellular compartments, or, if situated within the plasma membrane, across the cell surface into the extracellular space [8, 9].

Activation of the hypoxia inducible factor (HIF) family of transcription factors is one of the principle oxygen-responsive signaling pathways that allows the adaptation of cancer cells to hypoxia [2, 10]. Both prolyl hydroxylase domain (PHD) proteins and Factor Inhibiting HIF-1 (FIH-1) are oxygen sensors that control signaling through HIF [11]. PHDs hydroxylate HIF-1α, allowing Von Hippel Lindau (VHL) factor to bind, targeting HIF-1α for degradation. When oxygen levels decrease, the PHD proteins become inactive. Under these conditions, HIF-1α heterodimerises with HIF-1β and binds to hypoxic response elements (HREs) in target genes, leading to the expression of proteins that help hypoxic cancer cells survive [11]. While PHD is inactivated in hypoxia, FIH-1 retains its activity in low % O_2_ conditions [12, 13]. FIH-1 catalyzes the hydroxylation of the C-terminal transactivation domain (C-TAD) of HIF-1α, impairing the interaction between C-TAD and the co-activator proteins p300/CREB binding protein (CBP), leading to only partial HIF signaling [11]. FIH-1 can be inhibited by either severe hypoxic conditions [12], or membrane type-1 matrix metalloproteinase (MMP14) [14], a protein present on the plasma membrane of cells. FIH-1 inhibition through either of these mechanisms leads to p300/CREB binding to HIF-1α, resulting in full HIF-1 signaling.

It is widely believed that hypoxia and HIF have essential roles to play in cancer progression, as evidence indicates that altered cancer cell metabolism and HIF-regulated enzymes, such as CAIX, are crucial in the processes of tumor cell invasion and metastasis [15]. CAIX, NHE1 and V-ATPase, while contributing to the alkaline pH_i_ found in hypoxic cancer cells and enabling survival in their hostile environment, also lead to the formation of an acidic pH_e_. This acidosis supports cancer cell invasion through the degradation of the extracellular matrix (ECM), destabilizing intercellular contacts, and increasing the motility of cancer cells [16]. Hypoxic cancer cells, in addition to having an augmented capacity for invasion, are also more resistant to radiotherapy [17] and chemotherapy [1] than their aerobic counterparts, thereby contributing to breast cancer mortality [3].

The aberrant regulation of H^+^ ions, leading to a reversed pH gradient in cancer cells and tissues when compared with normal cells, is a phenomenon that is increasingly considered to be one of the most differential hallmarks of cancer [18]. The targeting of proteins in cancer cells that are responsible for the initiation/regulation of the reversed pH gradient may be selective for malignancy and could lead to the development of more effective and less toxic therapies against cancer, either alone through inhibiting invasion and metastasis or by augmenting the effects of radiotherapy/chemotherapy. The aim of this study was to assess the expression of CAIX, NHE1 and V-ATPase in breast cancer cells under differing O_2_ conditions, and to compare the therapeutic effect of targeting these proteins in both 2D and 3D breast cancer models.

## RESULTS

### Inhibitors of the pH regulating proteins CAIX, NHE1 and V-ATPase reduce the proliferation of breast cancer cells

**Figure 1:**
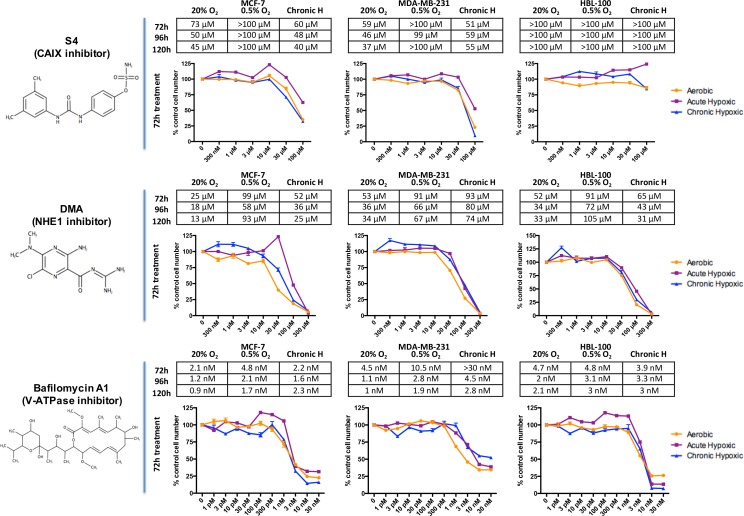
The effects of drugs targeting CAIX, NHE1 and V-ATPase on breast cancer proliferation in differing O_2_ conditions The effects of drugs targeting CAIX (S4), NHE1 (DMA) and V-ATPase (bafilomycin A1) on cancer cell proliferation were assessed through sulforhodamine B (SRB) assays. Breast cancer cells were treated with the inhibitors for 72, 96 and 120h in aerobic (20% O_2_) or acute hypoxic (0.5% O_2_) conditions. The effects of the drugs on the proliferation of chronic hypoxic cells, which were cultured for over 10 weeks in 0.5% O_2_ before treatment, were also evaluated. The tables show IC_50_ values for each of the inhibitors, the concentration of drug required to reduce proliferation by 50%. Graphs present representative data, showing the response of each of the cell lines to 72h drug treatment in aerobic, acute hypoxic and chronic hypoxic conditions, with each drug treated value normalized against a control value (bars represent SEM). Drug structures were drawn using MarvinSketch version 16.3.12, ChemAxon.

The effect of targeting pH regulator proteins on cancer cell proliferation was assessed through SRB assays. One of the drugs used, S4, has been shown to inhibit the tumor associated carbonic anhydrase IX and XII isoforms at low nanomolar levels, while having much less activity against the non-tumor associated cytosolic carbonic anhydrase isoforms [19]. It has been shown to be anti-metastatic against MDA-MB-231 lung metastases [19]. DMA (5-(N,N-Dimethyl)amiloride hydrochloride), used to target NHE1, and bafilomycin A1, used to inhibit V-ATPase, have also been demonstrated to be specific for their respective targets [20, 21]. Concentrations of the inhibitors found to be effective in previous studies were used [22–24]. Each cell line was treated with varying concentrations of drug and incubated for 72-120h in either 20% O_2_ or 0.5% O_2_ conditions. The effect of the inhibitors was also assessed on chronically hypoxic cells that had spent more than 10 weeks in 0.5% O_2_ before treatment (see Supplementary Figure 2). Acute hypoxic cells were placed into 0.5% O_2_ conditions 24h before treatment to allow them to adapt to hypoxic conditions, while the chronic hypoxic cells remained in 0.5% O_2_ conditions throughout. The data from these studies were used to calculate the IC_50_ (half maximal inhibitory concentration) for growth inhibition in response to all of the inhibitors in each of the cell lines (Figure 1).

These results demonstrate that all the inhibitors had anti-proliferative effects on the 3 breast cancer cell lines studied. The V-ATPase inhibitor bafiloymcin A1 was the most effective drug tested, with nanomolar concentrations having a large effect on cancer cell number. While hypoxic conditions and enhanced glycolysis would be assumed to increase the dependency of these cells on regulators of pH_i_, acute hypoxic cancer cells were more resistant to the anti-proliferative effects of each of the inhibitors, exhibiting raised IC_50_ values in comparison with the same cells in 20% O_2_. This increased resistance to drug treatment in acute hypoxic conditions was especially noticeable with the CAIX inhibitor S4, as at many of the time points in hypoxia no concentration of drug used (up to a concentration of 100 μM) led to a 50% reduction in cell number. In chronic hypoxia, MCF-7 and MDA-MB-231 cells had IC_50_ values for S4 that were similar to those seen in aerobic cells. HBL-100 cells were much more resistant to the effects of CAIX inhibition in both aerobic and hypoxic conditions compared to the other cell lines, with no concentration of S4 tested reducing cell number by 50%.

### The effect of hypoxia on expression of the target pH regulators

**Figure 2:**
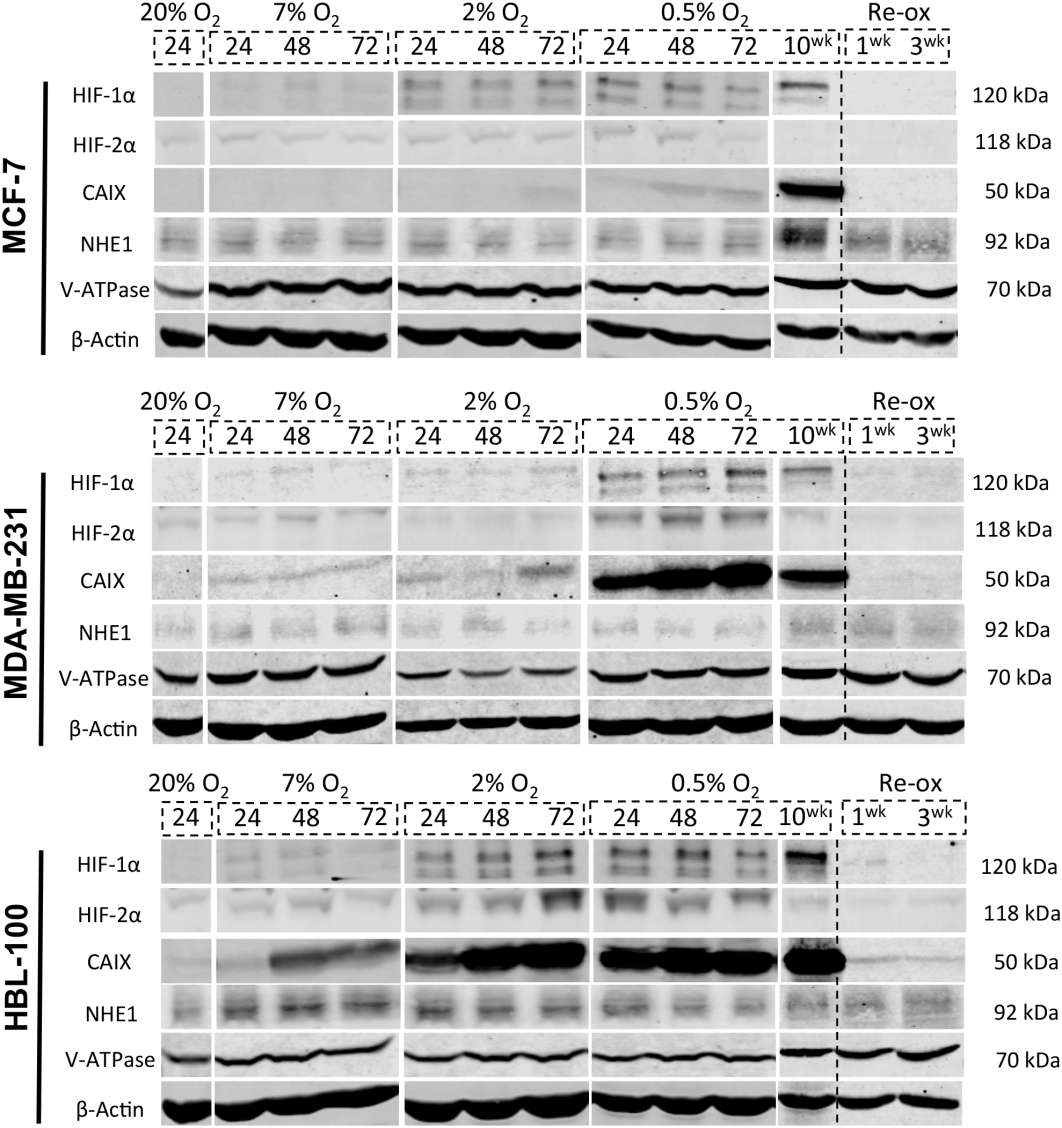
Target protein expression in varying oxygen concentrations in 2D Whole-cell lysates of MCF-7, MDA-MB-231 and HBL-100 cells were obtained to examine the levels of 5 target proteins (HIF-1α, HIF-2α, CAIX, NHE1, V-ATPase) in differing oxygen concentrations (20% O_2_, 7% O_2_, 2% O_2_ and 0.5% O_2_) at varying time points (24, 48 and 72h). Protein levels in cells cultured for 10 weeks in 0.5% O_2_ were also analyzed, as was expression in chronic hypoxic cells that were re-oxygenated for 1/3 weeks before lysate acquisition. Experiments looking at expression levels in the 10 week 0.5% O_2_ and 1/3 week re-ox lysates were conducted separately to the 24/48/72h westerns.

Because acute hypoxia increased resistance to the anti-proliferative effect of the inhibitors, protein expression levels of HIF-1α, HIF-2α, CAIX, NHE1 and V-ATPase were examined in MCF-7, MDA-MB-231 and HBL-100 cell lines cultured in differing O_2_ conditions for varying periods of time using Western blotting of whole cell lysates (Figure 2). All 3 cell lines exhibited increases in HIF-1α and HIF-2α expression in 0.5% O_2_, apart from MCF-7 cells, which did not show any change in HIF-2α levels in hypoxic conditions. Expression of the HIF-1α inducible gene CAIX varied between the cell lines, with MDA-MB-231 and HBL-100 cells expressing much larger amounts of CAIX compared to MCF-7 cells, which only markedly expressed CAIX under more chronic hypoxic conditions. NHE1 expression was unchanged across the 3 cell lines by the different oxygen conditions, with the exception of NHE1 levels observed in chronically hypoxic MCF-7 cells. An antibody targeting the ATP6V1A subunit of V-ATPase was used to assess V-ATPase expression; with this antibody, no changes in V-ATPase levels were observed in any of the cell lines cultured in the different O_2_ percentages. For each of the proteins analyzed, re-oxygenation of the chronic hypoxic cells led to target protein expression intensities returning to levels that were similar to those seen in aerobic cells, suggesting that such changes are reversible and non-permanent.

**Figure 3:**
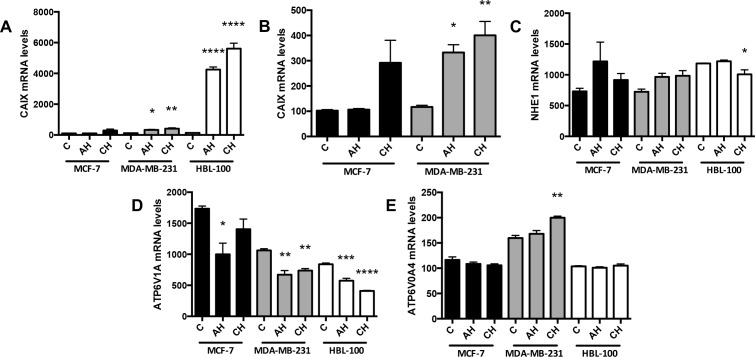
mRNA changes in response to different oxygen conditions CAIX **(A** and **B)**, **(B)** without the HBL-100 data), NHE1 **(C)** and V-ATPase subunits ATP6V1A **(D)** and ATP6V0A4 **(E)** mRNA levels were analyzed in cells cultured in 20% O_2_ (control, C), 0.5% O_2_ for 24h (acute hypoxia, AH) and 0.5% O_2_ for 10 weeks (chronic hypoxia, CH). Data expressed as mean ± SEM (n=3). *P≤0.05, **P≤0.01, ***P≤0.001 and ****P≤0.0001 (One-way ANOVA followed by Dunnett's multiple comparison test performed, comparing only the values within each cell line).

mRNA levels of the pH regulators were also assessed in aerobic, acute hypoxic and chronic hypoxic conditions. The CAIX mRNA results mirrored those of the CAIX protein in the 3 cell lines. There was a significant increase in CAIX mRNA levels in the MDA-MB-231 and HBL-100 cell lines cultured in acute and chronic hypoxic conditions (Figure 3A and 3B). MCF-7 cells in chronic hypoxia also exhibited increased CAIX mRNA levels compared to acute hypoxic or aerobic cells; this increase, however, was not significant (Figure 3B). No significant increases in NHE1 were observed in any of the cell lines (Figure 3C), while mRNA levels of the V-ATPase ATP6V1A subunit targeted in the westerns decreased in hypoxia (Figure 3D). Analysis of 2 other V-ATPase subunits, ATP6V0A3 and ATP6V0A4, which have been linked to the targeting of V-ATPase to the plasma membrane [25], showed that the mRNA levels of the ATP6V0A4 subunit significantly increased in hypoxic conditions in the MDA-MB-231 cell line (Figure 3E), whereas the mRNA levels of the ATP6V0A3 subunit stayed constant (Supplementary Figure 3).

### Reduced CAIX expression in MCF-7 cells is consistent with a higher activity of FIH-1 in this cell line

**Figure 4:**
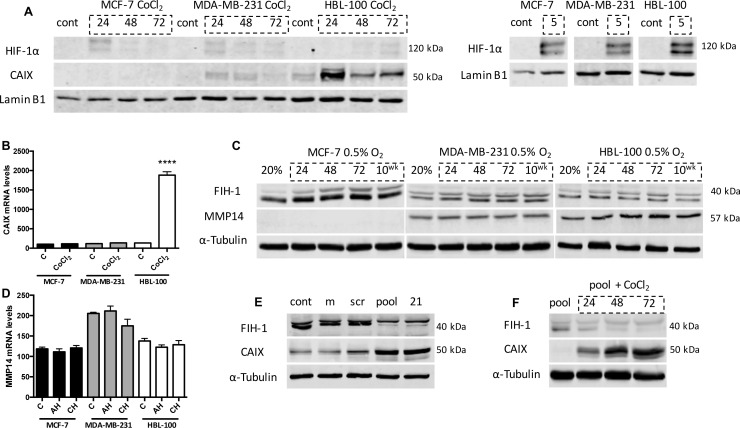
Investigating the cause of the differential expression patterns of CAIX between the cell lines **(A)** Nuclear levels of HIF-1α and cytoplasmic levels of CAIX in aerobic cells treated with 400 μM cobalt chloride (CoCl_2_) for 5, 24, 48 and 72h. Experiments assessing HIF-1α levels at 5h CoCl_2_ treatment were carried out separately to the other time points. **(B)** CAIX mRNA levels in aerobic cells (Control, C) and cells treated with 400 μM CoCl_2_ for 24h. ****P≤0.0001 (Unpaired t test, comparing each CoCl_2_ sample to the 20% O_2_ sample within each cell line). **(C)** Cytoplasmic FIH-1 (lower band) and MMP14 expression levels in both aerobic and hypoxic conditions in the 3 cell lines. **(D)** MMP14 mRNA levels in cells cultured in 20% O_2_ (C), 0.5% O_2_ for 24h (AH) and 0.5% O_2_ for 10 weeks (CH). **(E)** siRNA targeting FIH-1 was used to assess whether FIH-1 had any effect on CAIX expression in MCF-7 cells cultured in 0.5% O_2_ conditions. cont, control. m, transfection reagent alone. scr, scrambled siRNA. pool, mixture of 4 siRNAs targeting FIH-1. 21, single siRNA targeting FIH-1. **(F)** FIH-1 and CAIX expression levels in aerobic MCF-7 cells treated with either siRNA against FIH-1 alone, or both 400 μM CoCl_2_ and siRNA together for 24, 48 and 72h.

CAIX protein expression is induced by HIF-1α [13, 26]. Results showed that the 3 cell lines express very different amounts of CAIX, despite having similar levels of HIF-1α (Figure 2). To further analyze why CAIX expression varies, additional experiments were performed to assess CAIX levels in 20% O_2_ in the 3 cell lines when treated with cobalt chloride (CoCl_2_), which inhibits prolyl hydroxylase domain proteins (PHDs) and prevents the degradation of HIF-1α in aerobic conditions. All 3 cell lines had similar levels of stabilized HIF-1α when treated with CoCl_2_, with the largest amount of HIF-1α observed 5h after treatment in nuclear lysates (Figure 4A). However, CAIX protein expression again differed between the cell lines, with both CoCl_2_-treated MDA-MB-231 and HBL-100 cells expressing CAIX, while MCF-7 cells lacked a CAIX band in cytoplasmic samples (Figure 4A). These results were supported by mRNA analysis, which showed significantly higher CAIX mRNA levels in CoCl_2_-treated HBL-100 cells (Figure 4B) compared to un-treated cells. These findings suggest that the variation in CAIX proteins levels between these cell lines is not due to a disparity in HIF-1α expression.

FIH-1 is an oxygen sensor that inhibits HIF-1α C-TAD, thus reducing CAIX expression [27]. Membrane type-1 matrix metalloproteinase (MMP14) is a protein that inhibits FIH-1 [14]. To assess whether FIH-1 and MMP14 could be responsible for the differences in CAIX expression seen between these cell lines, protein levels were evaluated in lysates from the 3 cancer cell lines cultured in 0.5% O_2_ (Figure 4C). Results showed that MCF-7 cells had the highest levels of FIH-1 protein (lower band) and lacked MMP14. Both MDA-MB-231 and HBL-100 cells expressed slightly lower levels of FIH-1, while also expressing MMP14 (Figure 4C). Further analysis demonstrated that MCF-7 cells produce comparable levels of MMP14 mRNA to the other cell lines examined (Figure 4D). Therefore, differences in MMP14 protein levels observed were not due to a lack of gene transcription in MCF-7 cells. These results suggest that MMP14 and FIH-1 may co-operate to control CAIX expression in hypoxia in the cell lines.

To further assess whether FIH-1 was responsible for the low CAIX expression levels seen in MCF-7 cells, a mixture of 4 pooled siRNAs (Figure 4E, pool) and a single siRNA (Figure 4E, 21) targeting FIH-1 were used. Both led to reduced levels of FIH-1 in hypoxic MCF-7 cells compared to the controls, with concurrent increases in CAIX expression seen in these same samples (Figure 4E). Additionally, experiments were conducted using this siRNA to inhibit FIH-1 expression in aerobic MCF-7 cells that had HIF-1α stabilized as a result of CoCl_2_ treatment. Aerobic MCF-7 cells treated with both siRNA and CoCl_2_ together showed a strong induction of CAIX, while cells treated with siRNA alone did not express CAIX (Figure 4F). These data show that increased FIH-1 activity in MCF-7 cells contributes to the low CAIX expression seen in these cells when compared to the 2 other cell lines.

### The effect of S4 on the expression of pH regulating proteins

**Figure 5:**
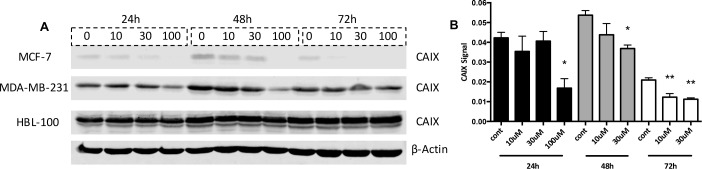
CAIX levels in S4-treated hypoxic cancer cells **(A)** Whole cell expression levels of CAIX were analyzed in the 3 cancer cell lines treated with the CAIX inhibitor S4. The cells were placed into 0.5% O_2_ conditions for 24h, and were then treated with different concentrations of S4 (10/30/100 μM) for 24, 48, and 72h. **(B)** CAIX expression levels (signal corrected for loading control) in S4-treated hypoxic MCF-7 cells. Data expressed as mean ± SEM (n=3). *P≤0.05, **P≤0.01 (One-way ANOVA followed by Dunnett's multiple comparison test performed, comparing only the values within each time point).

S4 treatment reduced the proliferation of human breast cancer cells lines in both aerobic and hypoxic conditions, with increased IC_50_ values observed in acute hypoxic cells (Figure 1). To examine whether these acute hypoxic cells had enhanced expression of the pH_i_ regulators in response to CAIX inhibition, thus facilitating resistance to this compound, western blots were performed on lysates from breast cancer cells treated with S4. Cells were cultured in 0.5% O_2_ for 24h to allow the cells to adapt to hypoxic conditions, after which the cells were treated with various concentrations of S4 for 24, 48 and 72h. MCF-7 cells were most sensitive to S4 treatment, as higher concentrations of the drug led to a significant reduction in CAIX levels at all time points (Figure 5A and 5B - 100 μM samples were not included at the later time points in B since at this concentration the drug affected cell number). While 100 μM concentrations of drug did reduce CAIX levels in MDA-MB-231 cells, this reduction was not found to be significant (Figure 5A). HBL-100 cells showed no loss of CAIX in response to S4 at any drug concentration (Figure 5A). Further, CAIX inhibition did not lead to any changes in either NHE1 or V-ATPase expression levels in any of the cell lines (Supplementary Figure 4).

### CAIX and NHE1 are up-regulated in hypoxic conditions in 3D

**Figure 6:**
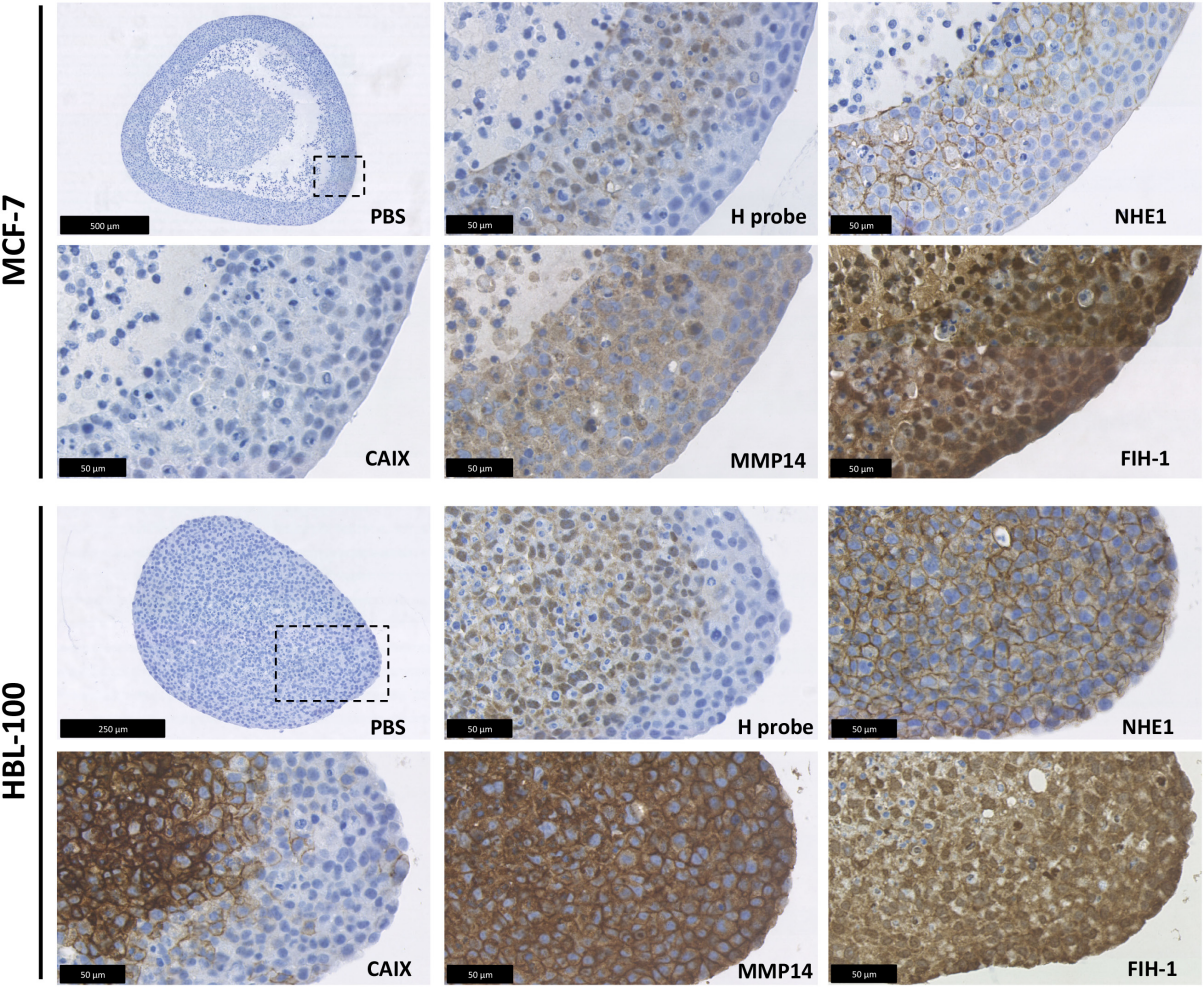

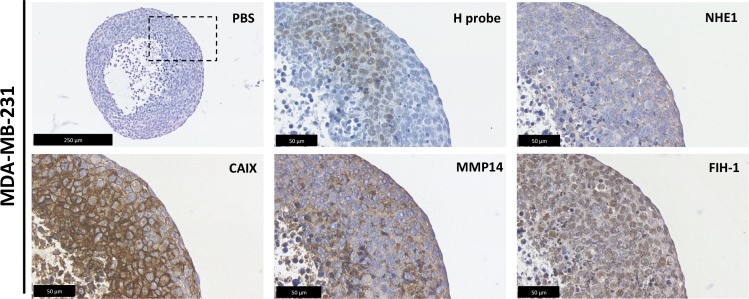
3D target protein expression in multicellular tumor spheroids 3D expression analysis was carried out in the MCF-7, MDA-MB-231 and HBL-100 cell lines using multicellular tumor spheroids. The spheroids were cultured in spinner flasks for 1 week before fixation. Hypoxyprobe was used to examine the formation of hypoxic areas within the spheroids. NHE1, CAIX, MMP14 and FIH-1 expression was also analyzed. Incubation for an hour with PBS instead of primary antibody acted as a control.

**Figure 7:**
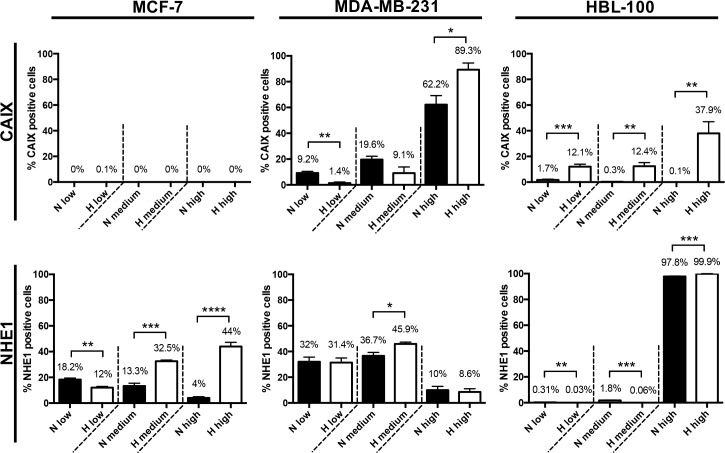
Quantitative analysis of CAIX and NHE1 protein expression levels in multicellular tumor spheroids Quantitative evaluation of CAIX and NHE1 protein expression levels within the normoxic and hypoxic regions of spheroids produced from MCF-7, MDA-MB-231 and HBL-100 cancer cell lines was performed using Definiens Architect XD 64 Tissue Studio 4.1. The percentage of cells exhibiting low, medium and high intensity levels of staining was calculated. Data expressed as mean ± SEM (n= at least 3 for each cell line). *P≤0.05, **P≤0.01, *** P ≤ 0.001, **** P ≤ 0.0001 (Unpaired t-tests performed).

Because 3D multicellular spheroids can more accurately replicate many aspects of the tumor microenvironment in comparison to cells cultured in 2D, including hypoxic and acidic gradients, the expression and location of several of the target proteins were investigated using this model (Figure 6). Quantitative evaluation of target protein expression was carried out, with hypoxyprobe used to delineate the normoxic and hypoxic areas within spheroids of each of the cancer cell lines. The percentage of cells with low, medium and high intensity levels of staining within the normoxic and hypoxic regions was analysed using Definiens Architect XD 64 Tissue Studio 4.1 (Figure 7 and Supplementary Figure 5). As in the 2D expression analysis, CAIX levels also differed between the cell lines when cultured in 3D. Strong CAIX staining was seen within the low % O_2_ areas of MDA-MB-231 and HBL-100 spheroids, with significantly increased percentages of cells with high intensity levels of CAIX staining present in the hypoxic regions of these spheroids (Figure 7). Only a small percentage of CAIX positive cells were detected within the hypoxic areas of MCF-7 spheroids. In contrast, strong induction of NHE1 protein expression was observed in the hypoxic regions of the MCF-7 spheroids, while NHE1 expression levels were comparable between the normoxic and hypoxic areas of spheroids of the 2 other cancer cell lines (Figure 7). V-ATPase expression was constant in both aerobic and hypoxic areas of spheroids in all of the cell lines (results not shown). MMP14 and FIH-1 expression was also assessed in 3D. While there were no differences in FIH-1 staining observed between the spheroids, MMP14 expression showed variation. Both MDA-MB-231 and HBL-100 spheroids exhibited large amounts of MMP14, with extensive plasma membrane staining visible in the HBL-100 cells. Cells within MCF-7 spheroids, however, had much lower levels of intracellular MMP14 staining. Examination of the 3D spheroid models demonstrate that while MCF-7 spheroids have little or no CAIX, they do express high levels of CAXII. This situation is reversed in both HBL-100 and MDA-MB-231 spheroids, where CAIX expression is higher, but CAXII is low (Supplementary Figure 6A). mRNA analysis of the 3 cell lines cultured in 2D in differing O_2_ conditions agrees with the 3D data, showing high CAXII mRNA levels in aerobic MCF-7 cells, with much lower levels of CAXII mRNA present in the aerobic MDA-MB-231 and HBL-100 cells (Supplementary Figure 7). These changes are contrasted with data for the reference gene – RPL32, which was unchanged across the sample set (Supplementary Figure 7).

### S4 treatment reduces the invasion of MDA-MB-231 cells in 3D

**Figure 8:**
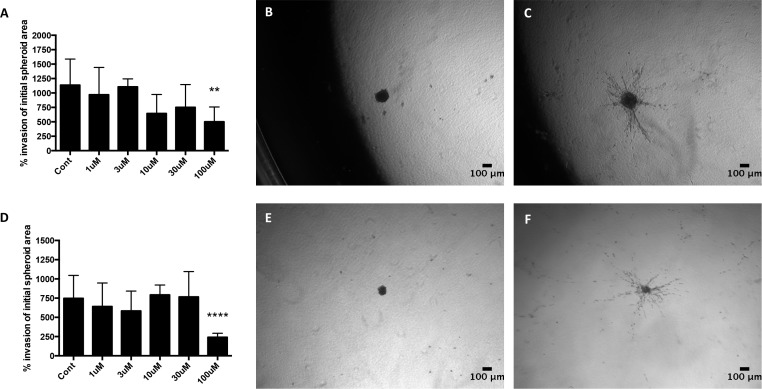
The effect of the CAIX inhibitor S4 on 3D cancer cell invasion from MDA-MB-231 spheroids in 20% O_2_ and 0.5% O_2_ conditions MDA-MB-231 spheroids were produced in spinner flasks, placed into collagen type 1 and left to invade for 48h in either 20% O_2_ or 0.5% O_2_ conditions. Invasion was measured using the image processing package FIJI. Graphs show the % invasion in 20% O_2_ **(A)** and 0.5% O_2_ **(D)** conditions. Data expressed as mean ± SD (n=4). **P≤0.01, ****P≤0.0001 (One-way ANOVA followed by Dunnett's multiple comparison test). Representative images of control spheroids present in collage type 1 in 20% O_2_ conditions for 0h **(B)** and 48h **(C)**, along with spheroids present in 0.5% O_2_ conditions for 0h **(E)** and 48h **(F)**, are shown.

pH plays a central role in tumor cell invasion and metastasis [16]. 3D invasion assays were carried out to compare the effects of CAIX, NHE1 and V-ATPase inhibition on cancer cell invasion from MDA-MB-231 and HBL-100 spheroids embedded in collagen type 1. MCF-7 spheroids were not used, as they do not invade in 3D culture. 100 μM S4 significantly reduced the invasion of cells from MDA-MB-231 spheroids, compared to un-treated spheroids, in both 20% O_2_ (Figure 8A) and 0.5% O_2_ cell culture conditions (Figure 8D). However the drug had no significant effect on the invasion of cells from HBL-100 spheroids in either aerobic or hypoxic conditions (Supplementary Figure 8). Drugs targeting NHE1 and V-ATPase had no inhibitory effect on the invasion of cells from either MDA-MB-231 or HBL-100 spheroids in either 20% O_2_ or 0.5% O_2_ conditions (Supplementary Figure 9 and 10). These results suggest that of these pH regulators, CAIX expression may be the most important in regard to facilitating invasion.

### CAIX and NHE1 inhibitors combine effectively with irradiation

**Figure 9:**
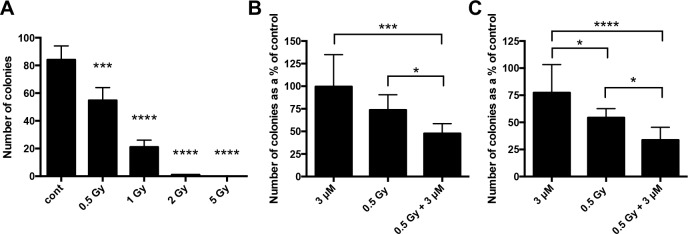
Clonogenic assays assessing the combination of drugs targeting CAIX and NHE1 with irradiation **(A)** MDA-MB-231 spheroids were treated with a range of radiation doses to see which dose would be optimal to assess any additive/synergistic drug effects. Data expressed as mean ± SD (n=3). *** P ≤ 0.001, **** P ≤ 0.0001 (One-way ANOVA followed by Dunnett's multiple comparison test). **(B)** 3D clonogenic assay performed with MDA-MB-231 spheroids treated with the CAIX inhibitor FC9403A. Data expressed as mean ± SD (n=8). *P≤0.05, ***P≤0.001 (Kruskal-Wallis test performed). **(C)** 3D clonogenic assay performed with MDA-MB-231 spheroids treated with the NHE1 inhibitor DMA. Data expressed as mean ± SD (n=9). *P≤0.05, ****P≤0.0001 (One-way ANOVA followed by Tukey's multiple comparison test).

Hypoxic cancer cells are relatively resistant to radiotherapy [17]. To investigate if drugs targeting pH regulatory molecules could increase the effects of irradiation, 3D clonogenic assays were performed with MDA-MB-231 spheroids. Figure 9A shows the response of cells from MDA-MB-231 spheroids to a range of irradiation doses. The low dose of 0.5 Gy was used in combination experiments to help observe additive or synergistic effects. The CAIX inhibitor S4 (Supplementary Figure 11A) and the V-ATPase inhibitor bafilomycin A1 (Supplementary Figure 11B) showed no combinatorial effects. However, studies with the more potent CAIX inhibitor FC9403A [22] (Figure 9B), and the NHE1 inhibitor DMA (Figure 9C), led to a significantly lower colony number when combined with irradiation compared against either irradiation or drug treatment alone.

**Figure 10:**
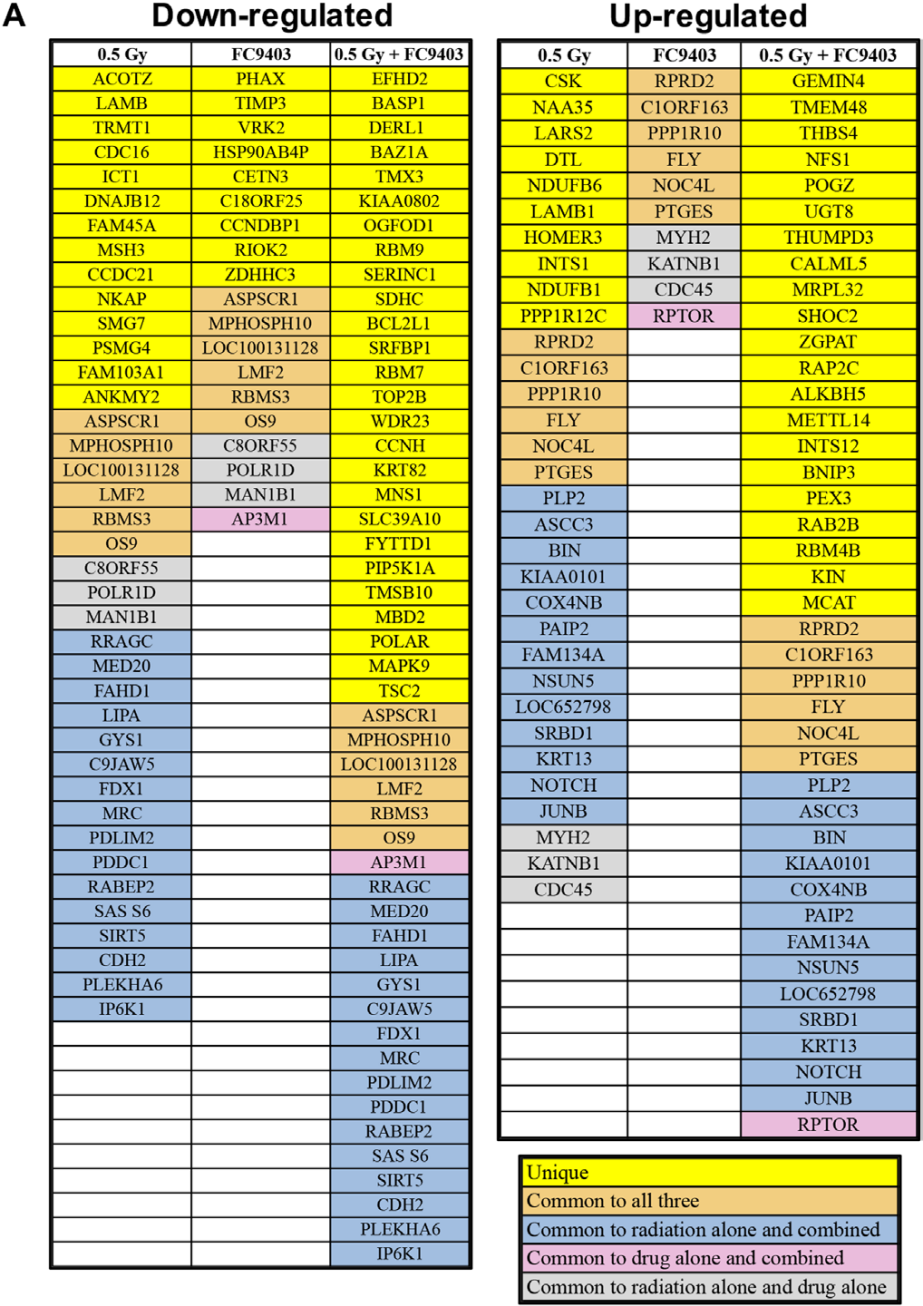

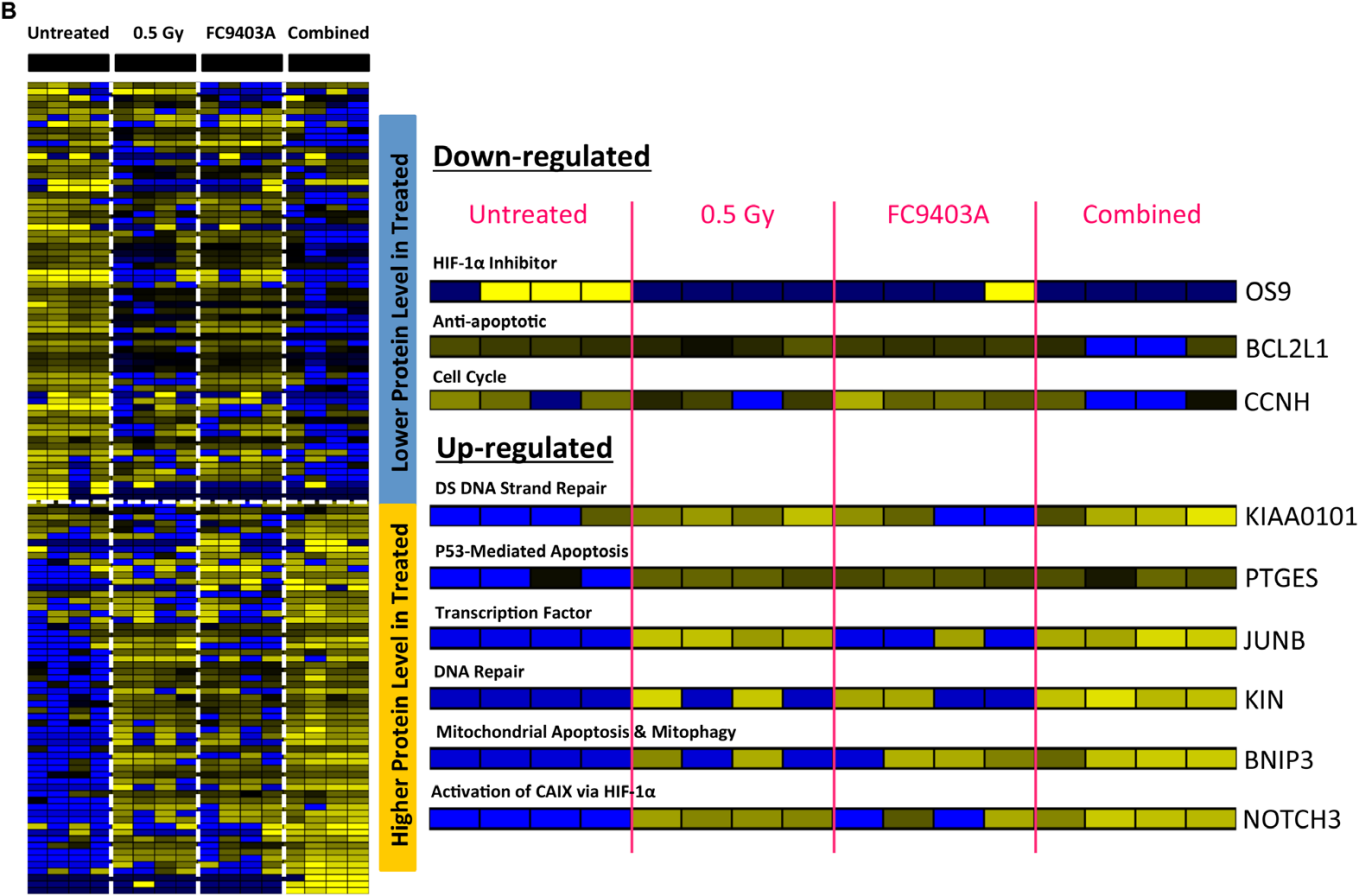
Proteomic-mass-spectrometric analysis assessing the mechanism of irradiation and CAIX inhibition combination Proteomic-mass spectrometric analysis was carried out on MDA-MB-231 cells treated with 0.5 Gy, 3 μM FC9403A, or a combination of both irradiation and CAIX inhibitor. Untreated MDA-MB-231 cells acted as the control. **(A)** Proteins identified as being either significantly up-regulated or down-regulated in the different treatment groups, identified using the Rank product test. **(B)** Protein changes detected within each of the different treatment groups, with examples of proteins with important biological functions highlighted.

Proteomic-mass-spectrometric analysis was carried out to analyze the possible mechanisms through which FC9403A was combining with irradiation in MDA-MB-231 cells. Overall, 5414 proteins were detected in this preliminary analysis. Proteins identified as being up-regulated or down-regulated in the different treatment groups are shown in Figure 10A, with proteins identified as having key biological functions indicated in Figure 10B (see Supplementary Figure 11C for the full list of proteins present in Figure 10B). The HIF-1α inhibitor OS9, the cell cycle regulator CCNH, and the anti-apoptotic BCL2L1 are examples of proteins that were significantly down-regulated in the cells that received a combination of both irradiation and drug. The pro-apoptotic molecules PTGES and BNIP3, the DNA repair proteins KIN and KIAA0101, the transcription factor JUNB, and the neural development signaling molecule NOTCH3 were all up-regulated in the combined treatment group.

## DISCUSSION

This study compared the therapeutic effect of targeting CAIX, NHE1 and V-ATPase in breast cancer, assessing the effects of inhibition in different O_2_ conditions using both 2D and 3D culture models. Inhibitors of each of the 3 targets demonstrated anti-proliferative effects in breast cancer cell lines (Figure 1 and Supplementary Figure 2), in agreement with other studies in breast and other cancer cell types [28–30]. The V-ATPase inhibitor bafilomycin A1 had the most potent effects on proliferation, reflecting the diverse biological roles performed by V-ATPase that are crucial for cell function [31].

Higher IC_50_ values for the inhibitors in acute hypoxia suggested increased resistance to the compounds in these conditions. These results seem counter-intuitive since it would be predicted that hypoxic cells, which are more reliant on glycolysis, would show amplified sensitivity to inhibition of pH regulating proteins. Resistance could be enhanced by increased target expression generated as cells adapt to acute hypoxia, meaning that higher concentrations of drug are needed in the hypoxic cells to induce the same effect seen in the aerobic cells. However, NHE1 showed little change in expression other than in MCF-7 cells in chronic hypoxia, while V-ATPase protein expression was unaffected by differing oxygen concentrations (Figure 2). Hypoxia can increase both CAIX and NHE1 activity, which may be a further factor in drug resistance [32–34]. Conversely, chronic hypoxic MCF-7 and MDA-MB-231 cells displayed similar IC_50_ values for the CAIX inhibitor to cells in aerobic conditions, suggesting that such resistance may be reversible.

Previous studies examining CAIX, NHE1 or V-ATPase expression in hypoxia produced disparate results [33–36]. In this study, both NHE1 protein and mRNA levels remained fairly constant in each of the cell lines under differing oxygen conditions, except for increased protein in chronically hypoxic MCF-7 cells (Figure 2), and a decrease in mRNA levels in HBL-100 cells cultured in chronic hypoxic conditions (Figure 3C). While no positive correlation between hypoxia and V-ATPase regulation has been shown to date [37], the distribution of V-ATPase within cells has been shown to be cell type dependent. For example, V-ATPase is present both intracellularly and on the cell surface in highly metastatic MDA-MB-231 cells, with lower levels found on the plasma membrane in non-metastatic MCF-7 cells [38]. V-ATPase subunit composition governs V-ATPase location within the cell. The ATP6V0A3 and ATP6V0A4 subunits are thought to target V-ATPase to the plasma membrane [25], where the protein regulates pH_i_ and contributes to invasion through the acidification of the extracellular environment. mRNA analysis of the ATP6V0A3 subunit showed that there was no significant differences in expression levels in hypoxic conditions (Supplementary Figure 3); however, significantly higher levels of the ATP6V0A4 subunit were expressed in chronically hypoxic MDA-MB-231 cells compared to cells in 20% O_2_ (Figure 3E), suggesting that chronic hypoxia may increase the levels of V-ATPase targeted to the plasma membrane in MDA-MB-231 cells. This would further account for the increased migratory capacity of this cell line in hypoxia [22].

HIF-1α and HIF-2α are both key mediators of the response of hypoxic cancer cells to low O_2_ conditions; HIF-1α responds to acute hypoxic conditions, while HIF-2α accumulates over time [39, 40]. These temporal responses were not observed in the cell lines that did express HIF-2α. In both MDA-MB-231 and HBL-100 cells, HIF-2α levels decreased in chronic hypoxic cells compared to acute hypoxic cells, while HIF-1α levels remained constant (Figure 2).

HIF-1α levels increased in hypoxic conditions in all cell lines examined. However, CAIX, which is induced by HIF-1 [13, 26], showed variable expression between cell lines and was only markedly up-regulated in MCF-7 cells after exposure to chronic hypoxia, implying that further mechanisms must be involved (Figure 2). While CAIX is a HIF-inducible gene, studies have shown that CAIX is only expressed when HIF-1α retains C-TAD activity (that is, when the co-activator p300/CBP is bound to HIF-1α) [27, 41]. HIF-1α gene expression can be inhibited by FIH-1, which hydroxylates the C-TAD of HIF-1α and prevents the binding of co-activators to HIF-1α [11]. MMP14 is a protein that can inhibit FIH-1 [14]. The active form of MMP14 is present on the cell surface as a 57 kDa transmembrane protein. Autocatalytic cleavage can generate a membrane-tethered inactive product (44 kDa) and a soluble catalytically inactive fragment (18 kDa) [42]. Westerns were performed looking at both FIH-1 and MMP14 levels in the 3 cell lines to assess whether these proteins might explain the differing CAIX results observed.

**Figure 11:**
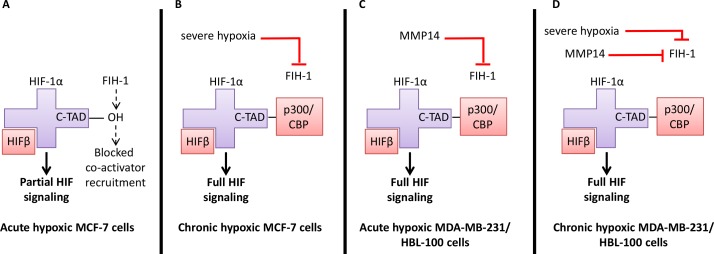
Regulation of HIF signaling in hypoxia and the consequences for CAIX expression in the 3 breast cancer cell lines **(A)** In acute hypoxic MCF-7 cells, FIH-1 has retained its activity. FIH-1 hydroxylates the C-TAD of HIF-1α, preventing co-activator binding. HIFβ binds to the HIF-1α that has been stabilized in hypoxia, resulting in partial HIF signaling and only a small amount of CAIX expression. **(B)** Severe hypoxia inactivates FIH-1, enabling the co-activator p300/CBP to bind to the C-TAD of HIF-1α, resulting in full HIF signalling and CAIX expression in chronic hypoxic MCF-7 cells. **(C)** FIH-1 activity may be reduced by MMP14 in acute hypoxic MDA-MB-231 and HBL-100 cells, enabling p300/CBP binding and full HIF signalling, leading to CAIX expression in acute hypoxic conditions in these cell lines. **(D)** Both severe hypoxia and MMP14 may act in combination to inactivate FIH-1 in chronic hypoxic MDA-MB-231 and HBL-100 cells, resulting in high levels of CAIX expression.

MCF-7 cells expressed the highest levels of FIH-1, but lacked active MMP14 (57 kDa) (Figure 4C). Therefore in acute hypoxic MCF-7 cells, FIH-1 can hydroxylate the C-TAD of HIF-1α, blocking the binding of p300/CBP and leading to only partial activation of HIF signaling. Although FIH-1 is still active at lower O_2_ percentages, severely hypoxic conditions have been shown to reduce activity [12], which may explain why MCF-7 cells can express CAIX under chronically hypoxic conditions (Figure 2). Conversely, MDA-MB-231 and HBL-100 cells expressed lower levels of FIH-1 but high levels of active MMP14 (Figure 4C). Therefore in acute hypoxic MDA-MB-231 and HBL-100 cells, MMP14 may inhibit FIH-1, enabling p300/CBP to bind to HIF-1α, leading to the expression of C-TAD sensitive genes such as CAIX. Prior reports have demonstrated that FIH-1 suppression through siRNA treatment leads to increased CAIX protein levels in in aerobic and acute hypoxic conditions in hepatoma and osteosarcoma cell lines [43]. In agreement with these results, siRNA targeting FIH-1 was seen to lead to increased levels of CAIX in both acute hypoxic and CoCl_2_-treated MCF-7 cells (Figure 4E and 4F). These observations are consistent with FIH-1 and loss of MMP14 being involved in the low CAIX expression levels seen in MCF-7 cells (illustrated in Figure 11). However, further work is required to confirm the role of MMP14 in the control of CAIX expression within these breast cancer cell lines.

Studies have shown that HIF-2α modulates MMP14 expression [44]. However, MMP14 protein was present in both MDA-MB-231 and HBL-100 cells in aerobic conditions when HIF-2α was absent, and although HIF-2α levels increased in hypoxic MDA-MB-231 and HBL-100 cells, MMP14 expression did not. All the cell lines transcribed similar levels of MMP14 mRNA (Figure 4D), suggesting that differences in gene expression cannot explain the lack of active MMP14 in MCF-7 cells. It is possible that these cells either do not translate the MMP14 protein, or do so in amounts that are below detectable levels. Otherwise, the MCF-7 cells may translate similar levels of MMP14 to the other 2 cell lines, but the protein may be rapidly broken down in 2D culture in MCF-7 cells.

Expression of the target molecules was also assessed in more physiological 3D tumor spheroid models. These studies demonstrated results similar to those observed in 2D culture. CAIX was induced in the hypoxic areas of HBL-100 spheroids, but little or no CAIX expression was apparent in the hypoxyprobe-positive areas of MCF-7 spheroids (Figures 6 and 7). CAIX expression was also evident within the hypoxic areas of the MDA-MB-231 spheroids, with some staining also present in cells outside the hypoxyprobe positive region. Because FIH-1 activity can be reduced under severely hypoxic conditions [12], and CAIX expression occurred in chronic hypoxic MCF-7 cells in 2D cultures, MCF-7 spheroids were further assessed for CAIX expression after 2 and 3 weeks of spheroid growth. However, no hypoxic cells were present in the 2/3 week MCF-7 spheroids (Supplementary Figure 12), preventing such analysis.

To understand whether similar processes governed the expression of CAIX in the spheroids, MMP14 and FIH-1 expression was analyzed. No differences in FIH-1 staining were observed between the different spheroids, but MMP14 expression did vary (Figure 6). Both MDA-MB-231 and HBL-100 spheroids exhibited large amounts of plasma membrane MMP14 staining, while only light intracellular staining was observed in MCF-7 spheroids. Because 2D analysis showed that MCF-7 cells do not express active 57kDa MMP14, we believe this staining may indicate the presence of the inactive 18kDa fragment of MMP14 produced after autocatalysis; firstly, because the antibody used binds to the extracellular domain of the protein and secondly, because the lack of CAIX staining in the MCF-7 spheroids suggests that FIH-1 is not inhibited and therefore any MMP14 present is possibly inactive. Further investigation into why both MDA-MB-231 and HBL-100 cells retain the active form of MMP14, while the MCF-7 cells do not, is merited.

The low expression of CAIX in both 2D and 3D MCF-7 models raises the question of why inhibitors such as S4 can inhibit proliferation in these cells. S4 has been shown to have high affinity for both the IX and XII isoforms of the carbonic anhydrase enzymes, with a *K*_I_ reported at 7 nM for CAIX and 2 nM for CAXII [19, 45]. Furthermore, CAXII is also a hypoxia-inducible protein [32]. Examination of the 3D spheroid models demonstrate that while MCF-7 spheroids have little or no CAIX, they do express high levels of CAXII. This situation is reversed in both HBL-100 and MDA-MB-231 spheroids where CAIX expression is higher, but CAXII is low (Supplementary Figure 6). Therefore S4 is most probably inhibiting the CAXII isoform of carbonic anhydrase in MCF-7 cells, leading to the anti-proliferative effects. mRNA analysis of the 3 cell lines cultured in 2D in differing O_2_ conditions agrees with the 3D data, showing high CAXII mRNA levels in aerobic MCF-7 cells, with much lower levels of CAXII mRNA present in the aerobic MDA-MB-231 and HBL-100 cells (Supplementary Figure 7). Interestingly, while the 2D protein (Figure 2) and mRNA (Figure 3) data show CAIX levels increasing in the hypoxic MCF-7 cells, the mRNA (Supplementary Figure 7) and protein (Supplementary Figure 6A and 6B) data show that CAXII levels decrease in hypoxic MCF-7 cells compared to aerobic cells, suggesting that CAXII expression may be responding to the increased CAIX expression in hypoxic conditions. Other studies have also shown a similar interaction between CAIX and CAXII expression [32, 46], as well as for CAIX and CAII [47]. Together, these results indicate that carbonic anhydrase isoform expression may depend on some form of interaction between the different isoforms.

3D invasion assays highlighted the ability of CAIX inhibitors to hinder the invasion of MDA-MB-231 spheroids, with 100 μM of S4 significantly reducing the amount of invasion in both aerobic and hypoxic conditions (Figure 8A and 8D). Because both active MMP14 and CAIX have been linked to invasion [22, 42], and MMP14 is connected to the expression of CAIX, our results may explain why MCF-7 cells and spheroids lack invasive capabilities. Although CAIX inhibition did not have any significant effects on the invasion of HBL-100 spheroids, further experiments assessing the effects of combining both CAIX and MMP14 inhibition may prove more effective.

Hypoxic cancer cells can tolerate a 2-3 times higher dose of irradiation than aerobic cells for the same effect [48]. Therefore, methods to improve the effectiveness of radiotherapy in hypoxic tumors are required. Radiotherapy in combination with chemotherapy has improved tumor response and patient survival, but the restricted specificity of current chemotherapy drugs used in combination can cause tissue toxicity [49]. Using inhibitors targeting proteins that are selectively expressed/activated in hypoxic cancer cells, such as the pH regulating proteins, has the potential to improve the treatment of patients. 3D clonogenic assays conducted with MDA-MB-231 spheroids demonstrated that the CAIX inhibitor FC9403A (Figure 9B) and the NHE1 inhibitor DMA (Figure 9C), in combination with irradiation, decreased colony formation in comparison to either treatment alone. Irradiation requires oxygen for effective therapy, therefore hypoxic cells at the center of the spheroids should show increased resistance to the effects of radiotherapy. While FC9403A produced a beneficial effect in combination with irradiation, S4 failed to do so (Supplementary Figure 11). The reasons for this are unclear, but we have previously observed that FC9403A was a more potent anti-invasive agent than S4 [22].

Proteomic-mass-spectrometric analysis was performed to investigate the possible mechanism of FC9403A combination with irradiation (Figure 10), with proteins identified as having key biological functions identified in this preliminary analysis (Figure 10B). The induction of apoptosis is one way in which radiotherapy achieves its therapeutic effect [50]. Cells that were treated with a combination of irradiation and FC9403A were shown to have higher levels of PTGES and BNIP3, both of which have been linked to the induction of apoptosis [51, 52] and lower levels of BCL2L1, which has been reported to have anti-apoptotic functions [53]. The induction of apoptosis after treatment with CAIX inhibitors has been shown in previous studies [22]. Therefore, one potential mechanism through which CAIX inhibition may be combining with irradiation could be through the stimulation of apoptotic cell death. Aside from apoptosis, radiotherapy can also lead to mitotic cell death as a result of the induction of DNA damage. DNA damage repair within cancer cells is key to deciding the response to irradiation treatment [54]. The combination of irradiation and FC9403A led to increased levels of KIN and HIAA0101, both of which have been linked to DNA repair [55, 56] suggesting that combination treatment may be increasing the extent of DNA damage induced.

Although previous studies support the benefits of combining irradiation with a molecular targeted agent, there remains a severe lack of clinical trials that test this strategy. The early evaluation of novel molecular targeted agents in combination with irradiation, as shown in this study, could lead to the identification of drugs that work effectively with irradiation, and improve patient survival [49]. Inhibitors targeting the pH regulating proteins that are selectively up-regulated/activated in hypoxic cancer cells, with their ability to also affect the invasion of cancer cells, could prove to be such agents. Of the three pH regulatory molecules, CAIX represents the target with the most promise, with one CAIX inhibitor (SLC-0111) currently in Phase I clinical trials.

## SUMMARY

This study compared the therapeutic effect of targeting CAIX, NHE1 and V-ATPase in both 2D and 3D breast cancer models. Inhibitors targeting these proteins were shown to have anti-proliferative effects on breast cancer cell lines. Expression analysis highlighted the up-regulation of two of the targets, CAIX and NHE1, in hypoxic conditions in both 2D and 3D, with the varying CAIX levels between the cell lines a result of differential FIH-1 activity, possibly resulting from varying MMP14 expression. The potential of pH inhibition as a possible treatment for cancer patients was further highlighted in the ability of drugs targeting CAIX to inhibit the invasion of cancer cells in 3D, while both CAIX and NHE1 inhibitors were shown to combine with irradiation in clonogenic assays. Proteomic-mass-spectrometric analysis indicated that CAIX inhibition might be combining with irradiation through stimulating apoptotic cell death.

## MATERIALS AND METHODS

### All materials were obtained from sigma-aldrich unless otherwise stated

### Cell culture

Breast cancer cell lines MCF-7, MDA-MB-231, and HBL-100 were cultured in DMEM (Gibco) supplemented with 10% FCS (fetal calf serum, PAA), 50 U ml^−1^ penicillin and 50 mg ml^−1^ streptomycin. Cell lines were obtained either from ATCC or PHE (Porton Down, Salisbury, UK) and were authenticated by STR profiling. All aerobic cells were incubated at 37°C with 5% CO_2_ in a humidified incubator. Hypoxic cells were cultured in a hypoxic chamber (Whitley H35 hypoxystation) and maintained at either 0.5%, 2% or 7% O_2_ and 5% CO_2_, balanced with N_2_ at 37°C.

### Pharmacological inhibitors

The V-ATPase inhibitor bafilomycin A1 was obtained from Wako Pure Chemical Industries, Ltd. The CAIX inhibitors S4 and FC9403A were synthesized by Fabrizio Carta and Claudiu Supuran.

### siRNA treatment

5 – 15 × 10^4^ MCF-7 cells were cultured for aerobic/hypoxic experiments in DMEM containing 10% FCS, with hypoxic cells cultured at 0.5% O_2_. After 24h, cells were transfected using Dharmafect transfection reagent (Dharmacon, T-2001-03), and a final siRNA concentration of 50 nM was used (pool [L-004073-03], single [L-004073-21], and scrambled [D-01810-01], Dharmacon). After 48h, the transfection reagents were replaced with DMEM + 10% FCS, and cells lysed 72h later. In aerobic MCF-7 CoCl_2_ experiments, CoCl_2_ was added at 24h intervals during this 72h period.

### Protein isolation

Whole cell lysates of aerobic or hypoxic cells were obtained after washing cultures with PBS and treating with lysis buffer (50 mM Tris pH 7.5, 5 mM EGTA pH 8.5 and 150 mM NaCl) containing one complete protease inhibitor tablet (Roche), 100 μl phosphatase inhibitor cocktail 2, 100 μl phosphatase inhibitor cocktail 3, 50 μl aprotinin and 1% Triton X-100. The samples were centrifuged at 13,000 g; all procedures were performed at 4°C.

Nuclear/cytoplasmic lysates were acquired by washing cells with PBS and spinning at 13,000 rpm for 1 min. The pellet was re-suspended in 400 μl of lysis buffer (10 mM HEPES pH7.8, 10 mM KCl, 2mM MgCl_2_, 0.1mM EDTA) for 15min before adding 15μl of a IGEPAL CA-630 solution. After vortexing, samples were spun at 13,000 rpm for 1 min. The supernatant contains the cytoplasmic fraction. 50 μl of solution containing 50 mM HEPES pH 7.8, 50 mM KCl, 300 mM NaCl, 0.1 mM EDTA and 10% sterile glycerol (VWR) was added and samples were rotated for 20 min at 4°C. Samples were centrifuged at 13,000 rpm for 5 min. The supernatant contained the nuclear fraction. All lysates were stored at -80°C. Protein concentration was determined using a BCA assay.

### Western blot analysis

Separated proteins were transferred onto an Immobilon-P transfer membrane (Millipore), which was blocked using a solution containing 50% PBS and 50% Odyssey Blocking Buffer (Li-Cor), before incubating overnight at 4°C with primary antibodies (ATP6V1A polyclonal antibody [Abnova, H00000523-A01, 1:2000], carbonic anhydrase IX antibody clone m75 [Bioscience Slovakia, 1:6000], purified mouse anti-NHE [BD Biosciences, 611774, 1:1000], HIF-1α [BD Biosciences, 610958, 1:250], HIF-2α [Novus Biologicals, NB100-122, 1:1000], Factor inhibiting HIF-1α antibody [Novus Biologicals, NB100-428, 1:1000], anti-MMP14 antibody [Abcam, ab51074, 1:4000]; anti-α tubulin [Abcam, ab7291, 1:10000], anti-β actin [Abcam, ab8227, 1:8000], and anti-Lamin B1 [Abcam, ab133741, 1:2500]. Signals were detected using IRDye 800CW [Li-Cor, 926-32210, 1:10000] and IRDye 680LT [Li-Cor, 926-68021, 1:10000] and a Li-Cor Odyssey Imager.

### SRB assay

Cells were seeded into 96-well plates and incubated overnight in 20% O_2_. Acute hypoxic cells were placed into the hypoxystation and cultured in 0.5% O_2_ for 24h (the chronic hypoxic cells were kept in 0.5% O_2_ at all times). Cells were drug-treated after 24h, and cultures fixed between 72-120h later by addition of 50 μl cold 25% TCA (trichloracetic acid) solution per well at 4°C for 1 h. Plates were washed with H_2_O. When dry, 50 μl SRB dye solution was added per well for 30 min. Plates were washed 4 times with 1% glacial acetic acid (VWR) and when dry, 150 μl Tris buffer solution was added per well. After 1 h, plates were read at 540 nM on a plate reader (BP 800 Biohit). IC_50_ values were calculated using the Excel package Fit Designer.

### Formation of the MTSs

Cell lines were grown as conventional 2D monolayer cultures to approximately 80% confluency before being trypsinized to yield single cell suspensions. These were transferred to spinner flasks (VWR) inside a humidified incubator of 5% CO_2_ at 37°C. Spheroids used in IHC were treated with pimonidazole HCI (Hypoxyprobe, HP1-100) for 1 h before fixation.

### Immunohistochemistry (IHC)

Formalin-fixed spheroids were embedded, cut and mounted on glass slides. The slides were de-paraffinized and antigens retrieved using a sodium citrate solution (0.1M citric acid, 0.1M NaCitrate). Endogenous peroxidase activity was inhibited with H_2_O_2_ solution (Dako), and non-specific background staining was blocked using Total Protein Block (Dako, X0909). Primary antibodies were incubated for 1h at room temperature (hypoxyprobe antibody [hypoxyprobe, HP1-100, 1:8000], carbonic anhydrase IX antibody clone m75 [Bioscience Slovakia, 1:1000], purified mouse anti-NHE [BD Biosciences, 611774, 1:400], FIH-1/HIF-AN antibody [Novus Biologicals, NB100-428, 1:1000], anti-MMP14 antibody [Abcam, ab51074, 1:250]). After 1h, 1 drop of envision labeled polymer (Dako, K4001/K4003) was added, and the slides were left for 30 min. DAB and substrate buffer (1:50) (Dako, K3466) were added to each section and slides counterstained in hematoxylin. Images of the slides were taken using the Nanozoomer XR (Hamamatsu). Quantitative evaluation of protein expression was performed with Definiens Architect XD 64 Tissue Studio 4.1.

### 3D invasion assays with MTSs

Cell matrix type 1-A (Alphalabs), 1:1000 acetic acid, 0.22 M NaOH, FCS and 10x DMEM were mixed on ice at concentrations of 25%, 45%, 10%, 10%, and 10% respectively to create a collagen gel. A single spheroid was taken up along with some of the collagen gel into a 0.5 ml volume, and placed into a well of a 24-well plate. Cultures were incubated for 1h to allow polymerization. A 200 μl pipette tip was used to loosen the collagen plug and 0.5 ml DMEM +/− drug was added. Photographs of MDA-MB-231 spheroids were taken at 0h and 48h, while HBL-100 were imaged at 0h and 72h, using phase contrast microscopy (Axiovert S100) (x5 objective). Invasion was measured using the image processing package FIJI [57].

### Irradiation experiments

Clonogenic assays were performed with MDA-MB-231 spheroids irradiated at 0.5 Gy using a Faxitron RX-650. After irradiation, the spheroids were dissociated and cells counted before re-plating 1 × 10^3^ in media with or without drug. The plates were left for a week, after which the cells were fixed and stained with 1, 9-dimethyl-methylene blue zinc chloride double salt. Only colonies of 50 cells or over were counted. Proteomic-mass spectrometric analysis was also performed with lysates that were acquired 24h after the initial treatment. The samples were subjected to FASP (Filter aided sample preparation) [58] and the resulting peptides cleaned up on stage tips [59] before analysis on a Thermo RSLC 3000 Nano with a home-packed 10 cm x 75 um, 1.8 um C18 particle, home-pulled packed emitter, coupled to QExactive Plus. 15 mins loading at 0.4 μL/min, 2% organic (ACN; 0.05% acetic acid) was followed by a 120 min linear gradient to 50% organic, and 80% wash. 2% equilibration completed the method. MS1 scan range was 300-1650, AGC 3×10^6^ and max ion time 60 ms. Fragmentation was data dependent, with AGC 2×10^4^, max ion time 250 ms, loop 12, top 12, and normalized collision energy of 26. Data were processed in MaxQuant version 1.5.3.17 [60] with uniprot human reference proteome (UP000005640_9606, release 2015_12), and normalized LFQ were generated with match-between-runs enabled. Initial analysis and QC was performed with R version 3.2.3 (2015-12-10) [61]. Raw data are available at the ProteomeXchange Consortium via the PRIDE partner repository with the dataset identifier PXD005706.

### mRNA expression studies

Pellets, comprising approximately 3×10^6^ cells, from cell lines (MCF-7, MDA-MB-231 and HBL-100), grown under the experimental conditions specified above, were collected by trypsinisation and stored at -70°C. RNA was extracted using the RNeasy Mini kit (Qiagen) and quantity and quality was verified on a Bioanalyser 2100 with RNA 6000 Nano Kit (Agilent) and Nanodrop 2000c (Thermo Scientific). RNA was reverse transcribed, amplified and labelled using the Illumina TotalPrep RNA Amplification kit (Ambion) and hybridised to whole genome HumanHT-12 v4 Illumina BeadChips. Arrays were scanned using an Illumina iScan. All kits were used according to the manufacturer's standard protocols. Raw gene expression files were filtered using the Illumina probe detection P-value then log2-transformed and quantile-normalised using the lumi Bioconductor package.

### Statistical analysis

ANOVAs followed by Dunnett's multiple comparison tests were performed to assess whether significant results were obtained in the drug treatment westerns and the invasion assays. Kruskal-Wallis tests and one-way ANOVAs followed by Dunnett's and Tukey's multiple comparison tests were performed for the clonogenic assays. Differentially expressed proteins were distinguished in the proteomic-mass-spectrometry analysis through rank product testing. GraphPad Prism 6 was used to perform the analyses.

## SUPPLEMENTARY MATERIALS AND FIGURES



## Abbreviations

AGC: (automatic gain control)
ATCC: (American Type Culture Collection)
ATP: (adenosine-5′-triphosphate)
BCA: (bicinchoninic acid)
CA: (carbonic anhydrase)
CBP: (creb binding protein)
CCNH: (cyclin H)
CoCl_2_: (cobalt chloride)
C-TAD: (C-terminal transactivation domain)
DAB: (3,3′-diaminobenzidine)
DMA: (dimethylamiloride)
DMEM: (Dulbecco's modified Eagle's Media)
ECM: (extracellular matrix)
EDTA: (ethylenediaminetetraacetic acid)
EGTA: (ethylene glycol-bis(β-aminoethyl ether)-N,N,N',N'-tetraacetic acid)
FASP: (filter aided sample preparation)
FCS: (fetal calf serum)
FIH-1: (Factor inhibiting HIF-1)
h: (hour)
HEPES: (4-(2-hydroxyethyl)-1-piperazineethanesulfonic acid)
HIF: (hypoxia inducible factor)
IHC: (immunohistochemistry)
IGEPAL: (octylphenoxypolyethoxyethanol)
JUNB: (Jun B Proto-Oncogene)
LFQ: (label-free quantitation)
mins: (minutes)
ms: (milliseconds)
NHE: (Na^+^-H^+^ exchanger)
PBS: (phosphate buffered saline)
PHD: (Prolyl hydroxylase domain)
PHE: (Public Health England)
PTGES: (Prostaglandin E Synthase)
SRB: (sulforhodamine B)
STR: (short tandem repeat)
V-ATPase: (vacuolar H^+^-ATPase).
