# Supplementary material for: Inhibition of pH regulation as a therapeutic strategy in hypoxic human breast cancer cells
Source: Oncotarget. 2017 Apr 17;8(26):42857–75. doi: 10.18632/oncotarget.17143 (PMC5522111; doi:10.18632/oncotarget.17143)
Supplement: Supplementary file 1 [file oncotarget-08-42857-s001.pdf]

Inhibition of pH regulation as a therapeutic strategy in hypoxic human breast cancer cells

SUPPLEMENTARY DATA

SUPPLEMENTARY FIGURES

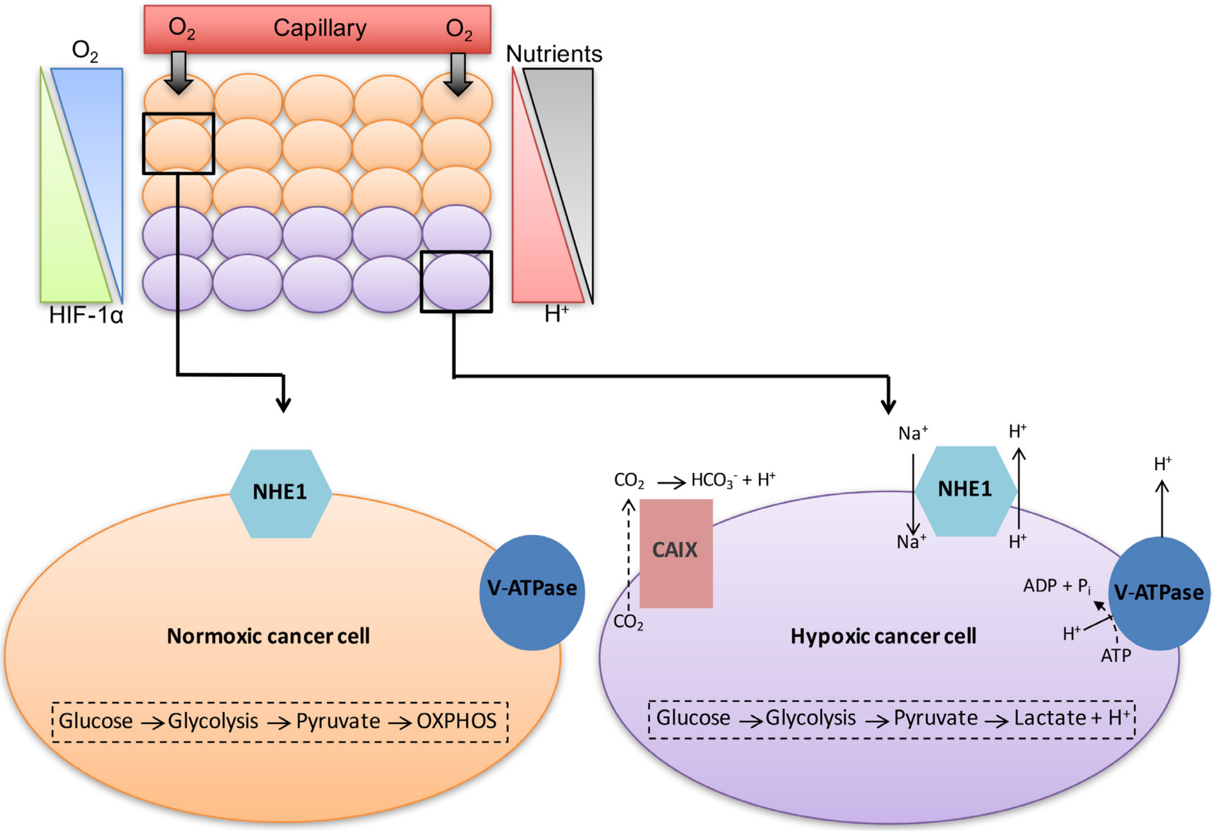

Supplementary Figure 1: Hypoxia, HIF and pH. As cancer cells are pushed further away from blood vessels, the level of O_2 and nutrients available to the cells decreases. Normoxic cells produce energy through glycolysis followed by oxidative phosphorylation; however, hypoxic cancer cells are unable to do this due to low O_2 levels. Activation of the hypoxia inducible factor (HIF) family of transcription factors is one of the principle oxygen-responsive signaling pathways that allows the adaptation of hypoxic cancer cells to this hostile microenvironment. HIF signaling shifts energy production in cancer cells from oxidative phosphorylation in the mitochondria towards glycolysis, allowing hypoxic cancer cells to continue to produce energy despite the low O_2 levels. However, this increased dependency on glycolysis leads to the production of increased amounts of H^+ ions. To help deal with the excess H^+ ions being produced, hypoxic cancer cells up-regulate/activate a number of pH regulating proteins; these proteins include CAIX, NHE1 and V-ATPase.

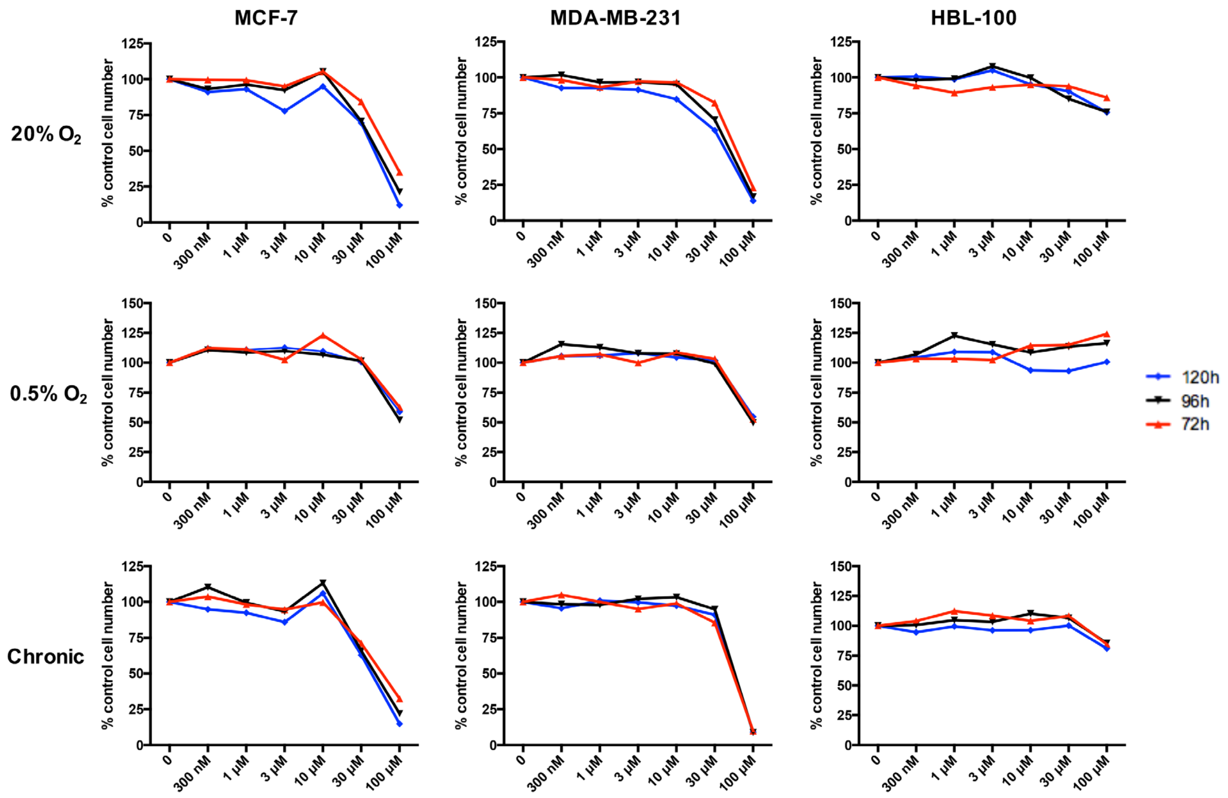

Supplementary Figure 2: SRB assay graphs showing the response of the 3 breast cancer cell lines to S4 in differing O_2 conditions. SRB assays were performed to assess the effects of drug treatment on cancer cell proliferation. Graphs show the response of MCF-7 (left column), MDA-MB-231 (middle column) and HBL-100 (right column) cells to S4 in 20% O_2 (top row) and acute hypoxia (middle row). The effects of S4 on the proliferation of chronic hypoxic cells, which had spent over 10 weeks in 0.5% O_2 conditions before drug treatment, were also measured (bottom row).

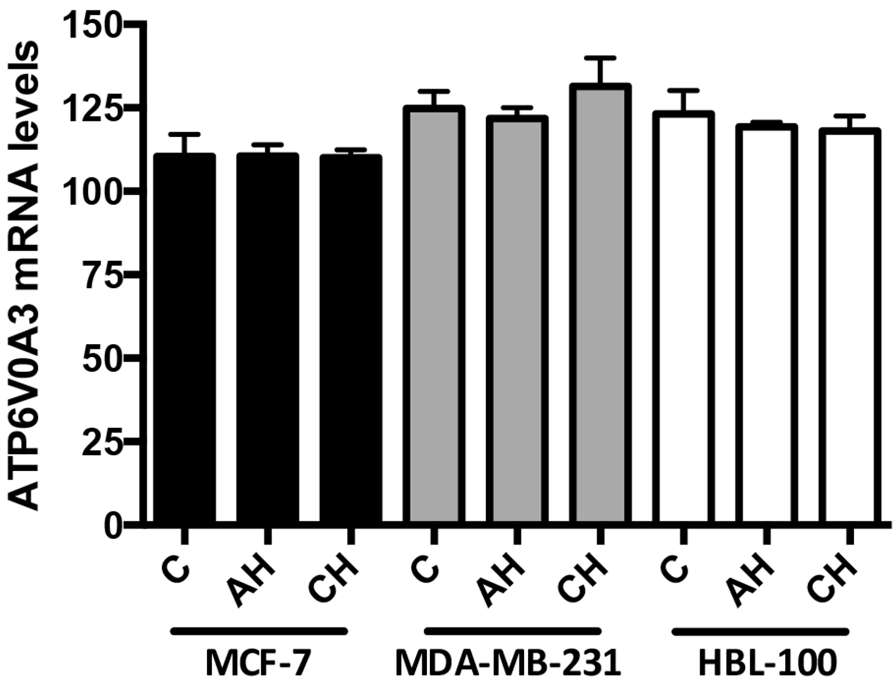

Supplementary Figure 3: ATP6V0A3 mRNA levels in differing O_2 concentrations. ATP6V0A3 mRNA levels were analyzed in cells cultured in 20% O_2 (C), 0.5% O_2 for 24h (AH) and 0.5% O_2 for 10 weeks (CH). Data expressed as mean ± SEM (n=3). No significant differences were found (One-way ANOVA followed by Dunnett's multiple comparison test performed, comparing only the values within each cell line).

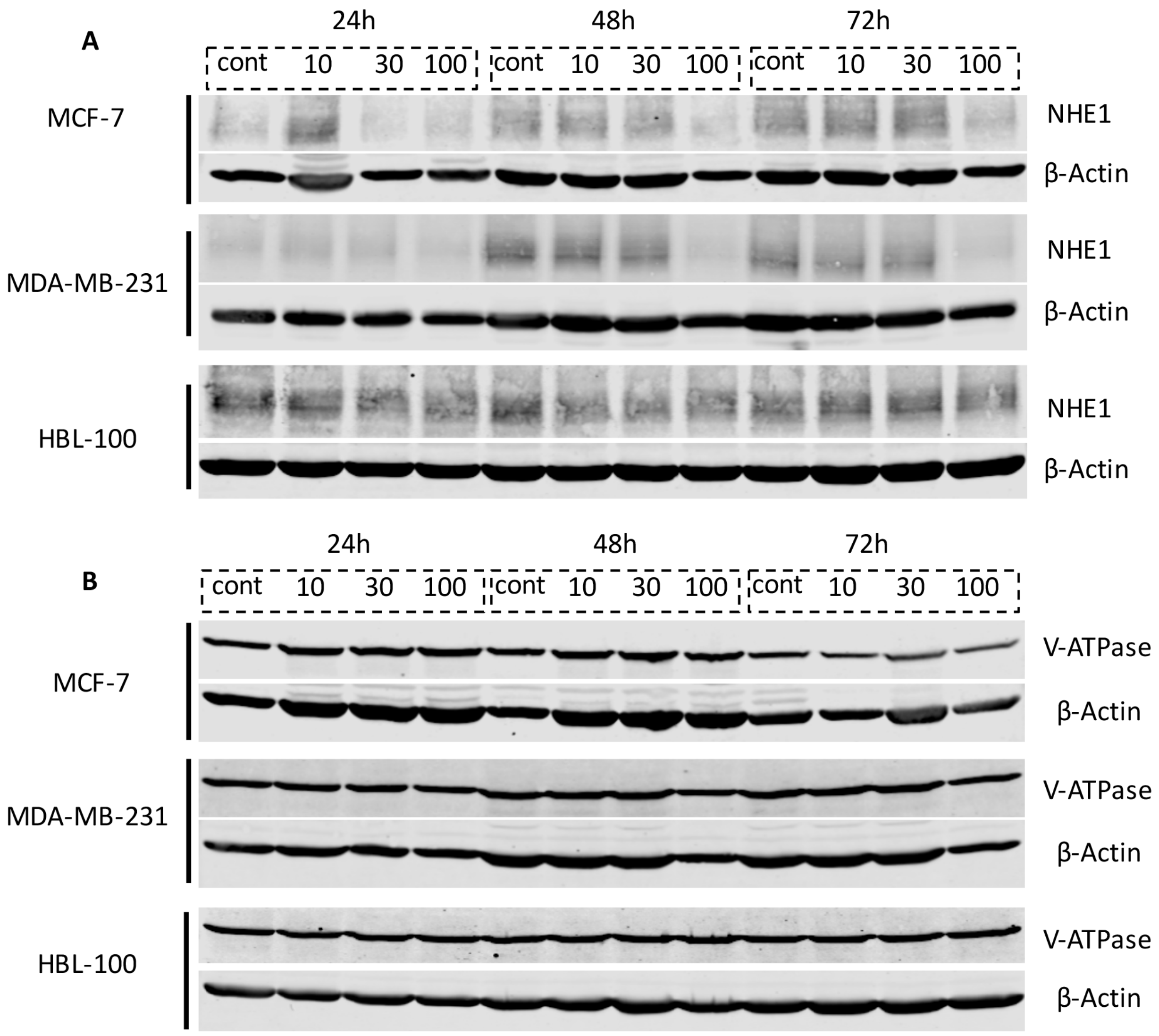

Supplementary Figure 4: NHE1 and V-ATPase expression levels in hypoxic S4-treated cancer cells. **(A)** MCF-7, MDA-MB-231 and HBL-100 cells were cultured in 0.5% O_2 conditions for 24h, after which they were treated with different concentrations (10, 30 and 100 μM) of the CAIX inhibitor S4 for 24, 48 and 72h. Untreated cells cultured in 0.5% O_2 acted as controls. Western blotting was performed to detect NHE1 **(A)** and V-ATPase **(B)**, with β-Actin used as the loading control.

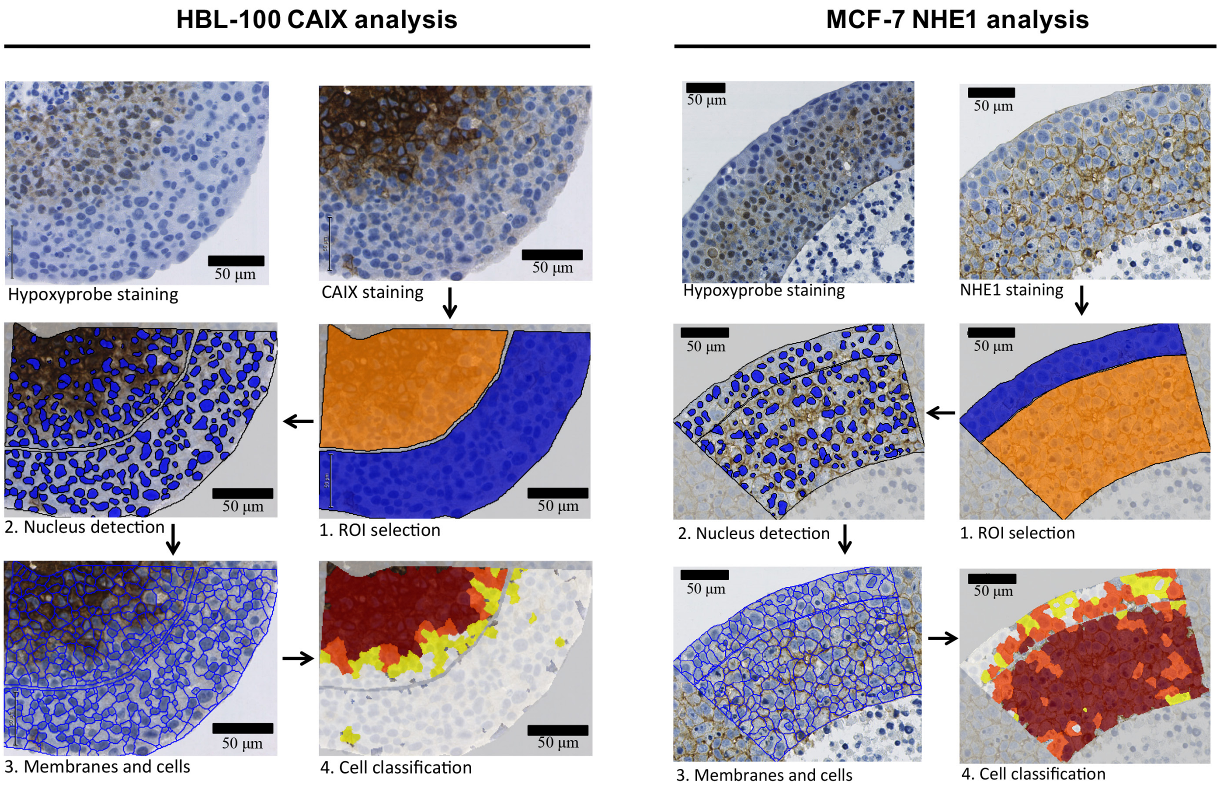

Supplementary Figure 5: Quantitative analysis of CAIX and NHE1 protein expression levels. Protein expression levels of CAIX and NHE1 were analysed using Definiens Architect XD 64 Tissue Studio 4.1. A combination of different 'cellular analysis actions' available in the software were used. (1) Areas encompassing the normoxic and hypoxic areas of the spheroids were selected. (2) Nucleus detection action was used to detect the presence of nuclei. (3) Membranes and cells action was used to simulate a cell body using the presence of nuclei and plasma membrane staining. (4) Cell classification action sub-classified each of the cells based on the intensity of plasma membrane staining (yellow, low intensity; orange, medium intensity; red, high intensity; white, no staining).

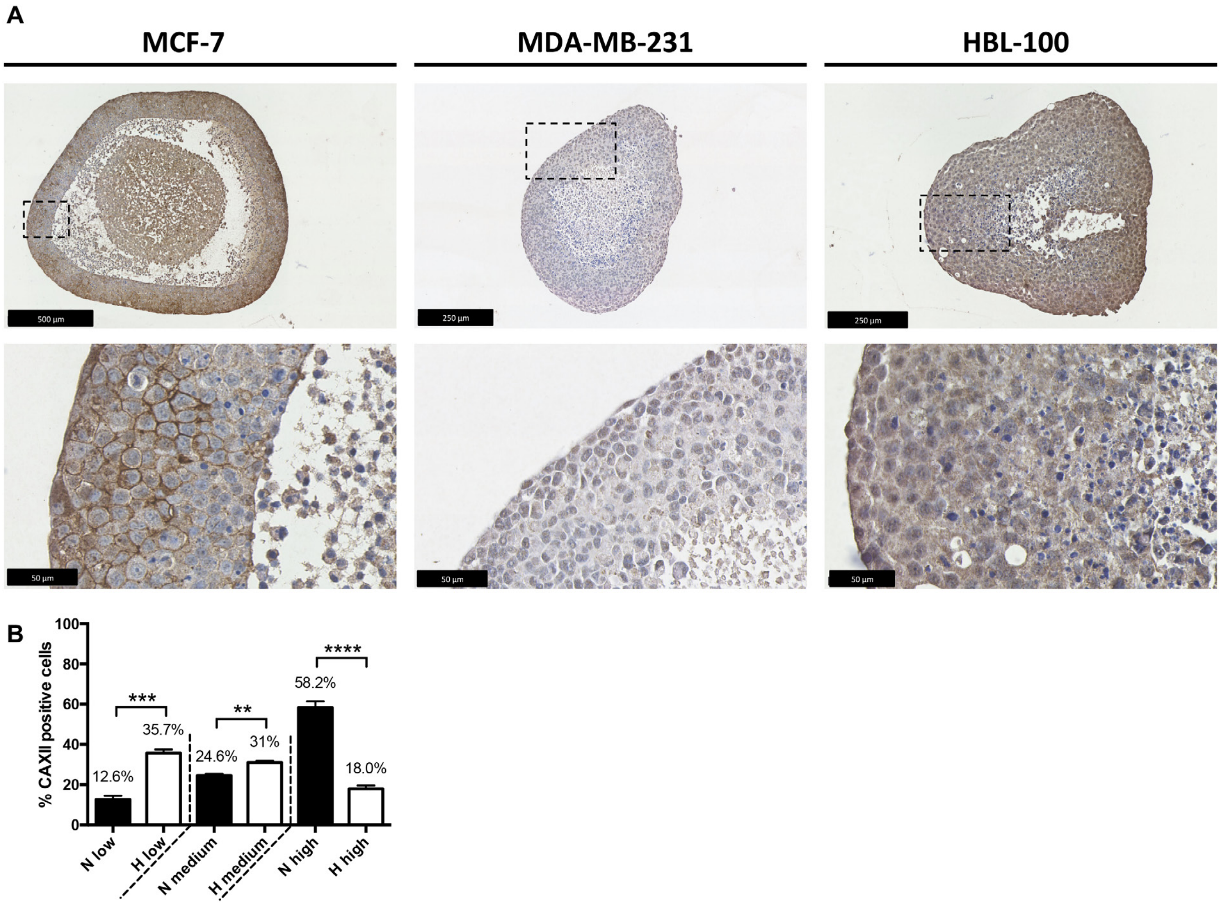

Supplementary Figure 6: 3D CAXII expression in spheroids. **(A)** CAXII expression analysis was carried out in the MCF-7, MDA-MB-231 and HBL-100 multicellular tumor spheroids. **(B)** Quantitative evaluation of CAXII protein expression levels within the normoxic and hypoxic regions of MCF-7 spheroids, showing the percentage of cells exhibiting low, medium and high intensity levels of staining. Data expressed as mean ± SEM (n=4). ** P≤0.01, *** P ≤ 0.001, **** P ≤ 0.0001 (Unpaired t-tests performed).

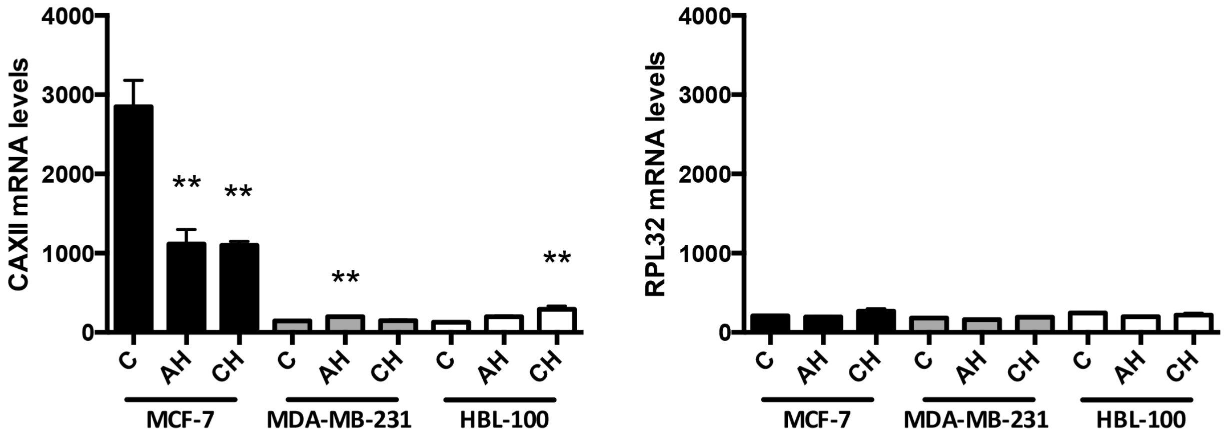

Supplementary Figure 7: CAXII mRNA levels in differing O_2 concentrations. CAXII mRNA levels were analyzed in cells cultured in 20% O_2 (control, C), 0.5% O_2 for 24h (AH) and 0.5% O_2 for 10 weeks (CH). Data expressed as mean ± SEM (n=3). **P≤0.01 (One-way ANOVA followed by Dunnett's multiple comparison test performed, comparing only the values within each cell line). Data for the reference gene RPL32 is also shown for comparison.

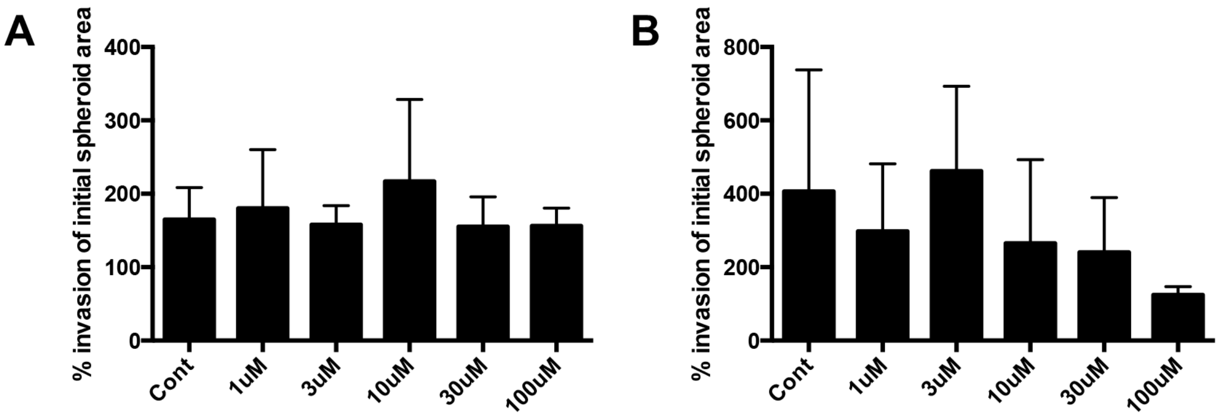

Supplementary Figure 8: The effect of S4 on 3D cancer cell invasion from HBL-100 spheroids in 20% O_2 and 0.5% O_2 conditions. The effects of S4 on the invasion of HBL-100 cells in 3D. HBL-100 spheroids were produced in spinner flasks, placed into collagen type 1 and left for 72h to invade in either 20% O_2 **(A)** or 0.5% O_2 **(B)**. % invasion was measured using the image processing package FIJI. Data expressed as mean ± SD (n=4). No significant differences in invasion were seen (One-way ANOVA followed by Dunnett's multiple comparison test).

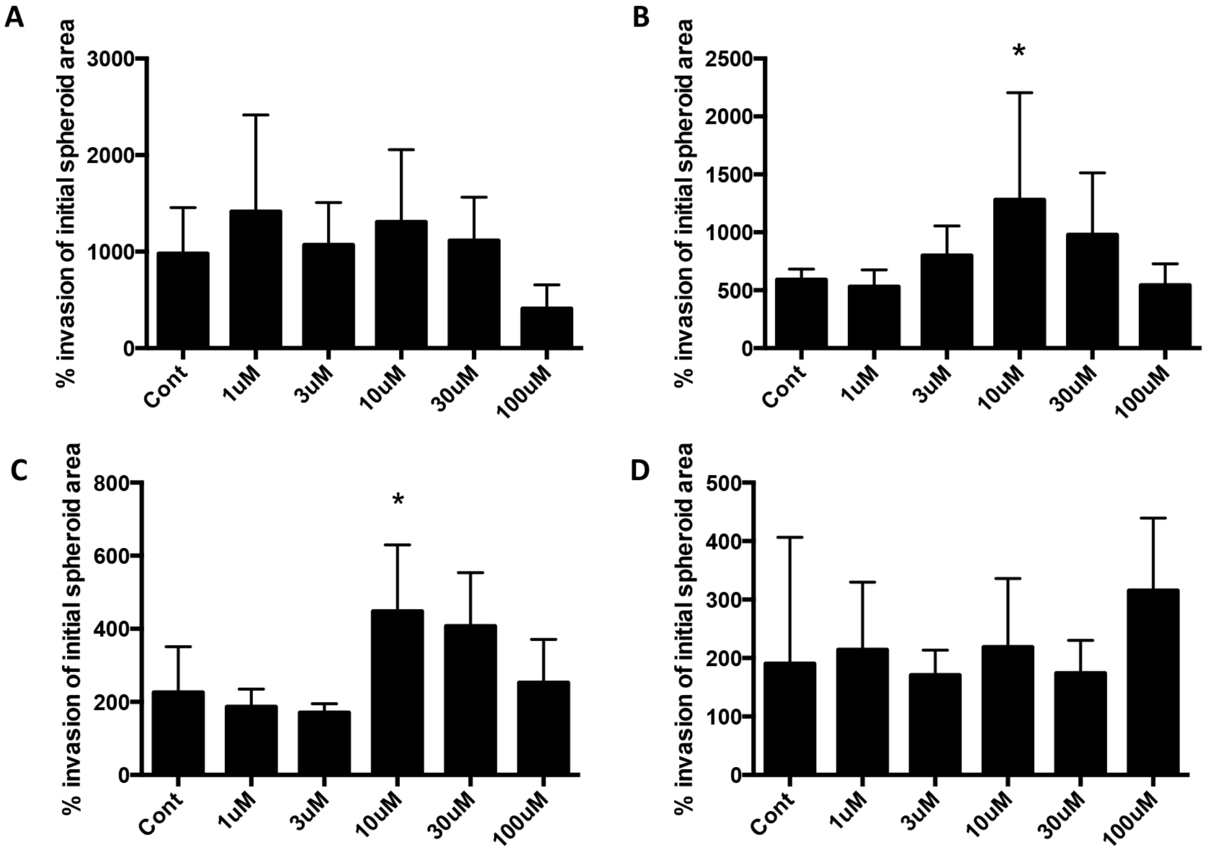

Supplementary Figure 9: The effect of the NHE1 inhibitor DMA on 3D cancer cell invasion from MDA-MB-231 and HBL-100 spheroids in 20% O_2 and 0.5% O_2 conditions. The effects of the NHE1 inhibitor DMA on cancer cell invasion from MDA-MB-231 spheroids in 20% O_2 **(A)** and 0.5% O_2 **(B)**, and HBL-100 spheroids in 20% O_2 **(C)** and 0.5% O_2 **(D)** conditions, was measured in 3D invasion assays. % invasion was measured using the image processing package FIJI. Data expressed as mean ± SD (n=4). *P≤0.05 (One-way ANOVA followed by Dunnett's multiple comparison test).

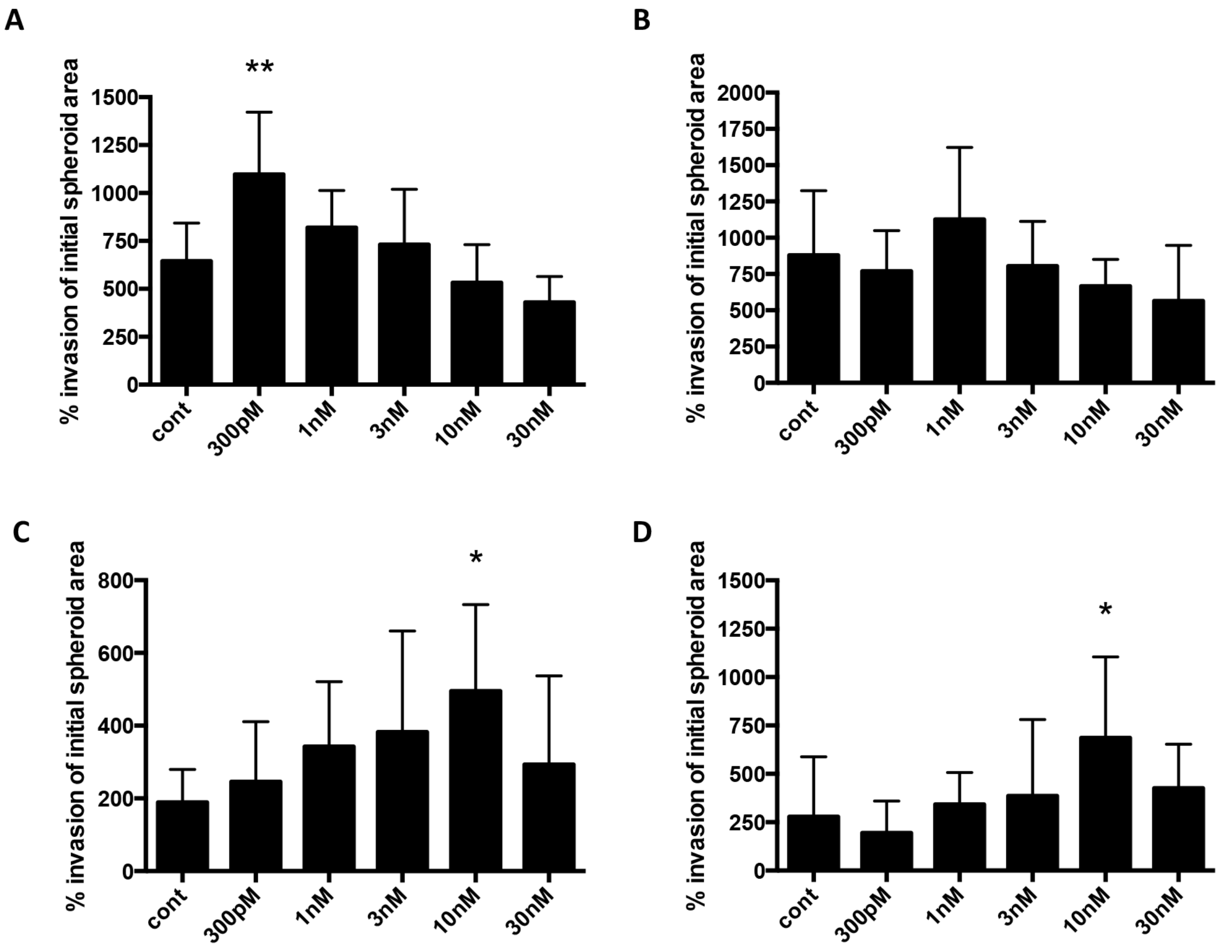

Supplementary Figure 10: The effect of the V-ATPase inhibitor bafilomycin A1 on 3D cancer cell invasion from MDA-MB-231 and HBL-100 spheroids in 20% O_2 and 0.5% O_2 conditions. The effects of the V-ATPase inhibitor bafilomycin A1 on cancer cell invasion from MDA-MB-231 spheroids in 20% O_2 **(A)** and 0.5% O_2 **(B)**, and HBL-100 spheroids in 20% O_2 **(C)** and 0.5% O_2 **(D)** conditions, was measured in 3D invasion assays. % invasion was measured using the image processing package FIJI. Data expressed as mean ± SD (n=4). *P≤0.05, **P≤0.01 (One-way ANOVA followed by Dunnett's multiple comparison test).

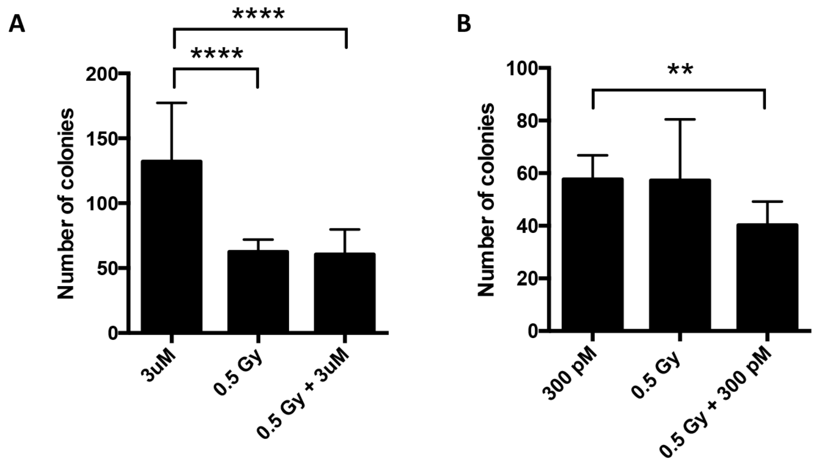

C

C1BORF25	CLIC3
C4ORF14	ACOT7
PIGT	ICT1
DERL1	CBORF55
SLC39A10	KCTD10
NMT2	EXTL2
SMURF2	PEG10
RBM7	TGFB2
FYTTD1	CENPB
PAK1TP1	TACC1
CDC16	MTX1
ELMOD2	RPTOR
POLR1D	RPRD2
RBM9	LCMT2
MED20	VRK2
FAM45A	FBXL18
OS9	MTPAP
BCL2L1	TMEM48
TMSB10	HAX1
MNS1	UGT8
RRAGC	BTN1
FDX1	KIAA0101
PLEKHA6	KRT13
PIP5K1A	PTGES
SERINC1	SRBD1
EFHD2	LOC652798
BAZ1A	PAIP2
BASP1	INTS12
GYS1	FAM134A
MPHOSPH10	PEX3
SASS6	ASCC3
RABEP2	FLG
OGFOD1	POGZ
PLD3	ZGPAT
ASPH	SHOC2
MRC2	METTL14
YES1	RBM4B
TMX3	KIN
FDLIM2	COX4NB
TXLNA	JUNB
LIPA	PLP2
CGGBP1	PPP1R10
TF	RAP2C
RPL6P27	ACAP2
TRIM26	MCAT
SIRT5	NFS1
CDH2	C1ORF163
SDHC	NOC4L
POLA2	BNIP3
MAPK9	RAB2B
ASPSCR1	THB34
LMF2	NDUFAP3
AP3M1	MRPL32
C9JAW5	THUMPD3
TSC2	NSUN5
KIAA0802	NOTCH3
MBD2	ALKBH5
LOC10013112B	CALML5
TOP2B	MED1
CCNH	CDC42EP2
WDR33	TNRC6B
KRT82	
GNB1L	
RBNS3	
IP6K1	

Supplementary Figure 11: Irradiation experiments targeting the different pH regulatory proteins. **(A/B)** 3D clonogenic assay performed with MDA-MB-231 spheroids treated with S4 **(A)** and bafilomycin A1 **(B)**. Data expressed as mean ± SD (n=9). **P≤0.01, ****P≤0.0001 (One-way ANOVA followed by Tukey's multiple comparison test). **(C)** List of proteins identified as being down-regulated (blue) and up-regulated (orange) in the treated samples present in Figure 10B.

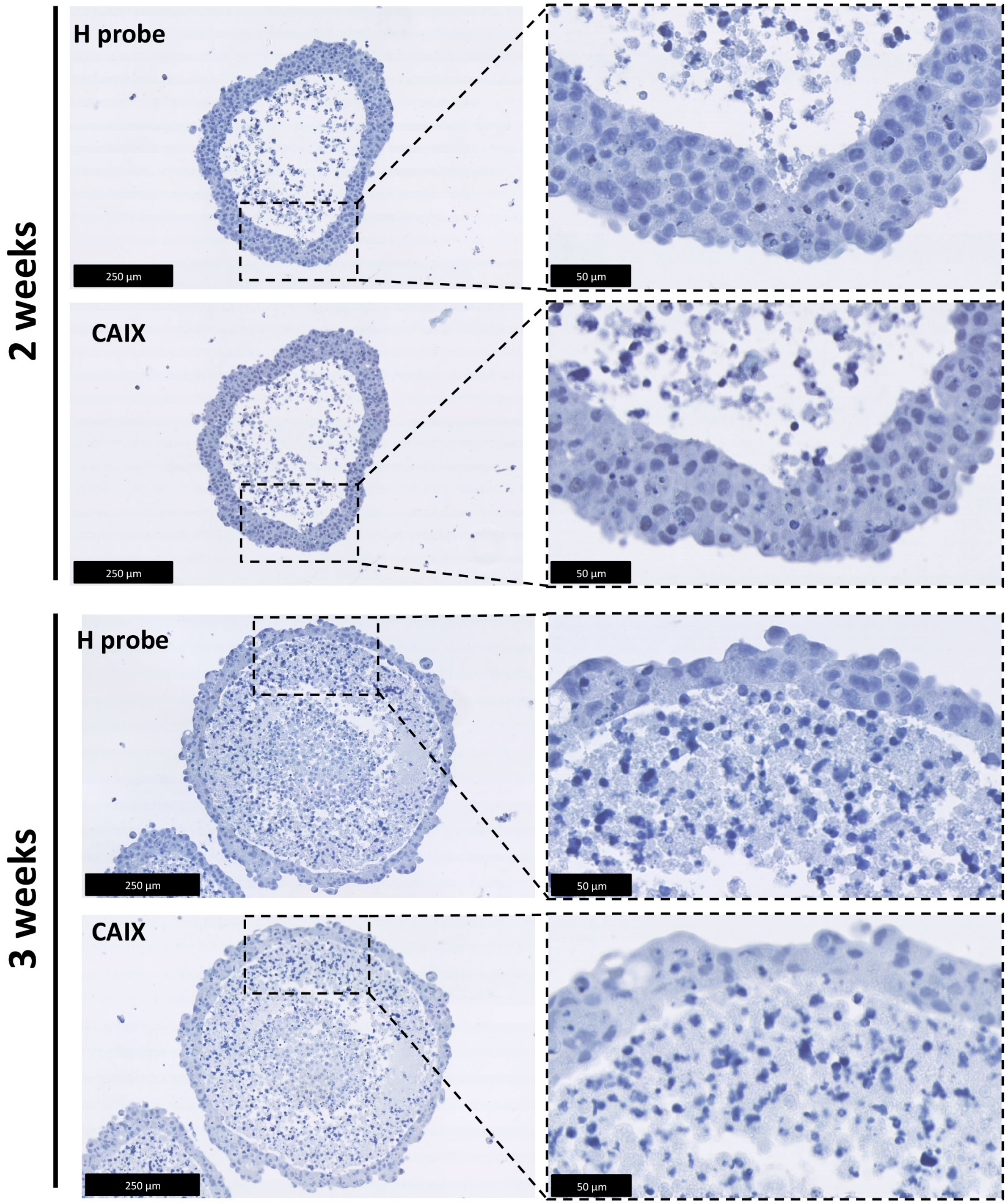

Supplementary Figure 12: Hypoxyprobe and CAIX staining in 2 and 3 week old MCF-7 spheroids. 3D expression analysis was carried out in the MCF-7 cells using spheroids that were cultured for 2 and 3 weeks before fixation. IHC was performed, analyzing both hypoxyprobe and CAIX staining.
